# Insights into the genomic and functional divergence of *NAT* gene family to serve microbial secondary metabolism

**DOI:** 10.1038/s41598-024-65342-4

**Published:** 2024-06-28

**Authors:** Sotiria Boukouvala, Evanthia Kontomina, Ioannis Olbasalis, Dionysios Patriarcheas, Dimosthenis Tzimotoudis, Konstantina Arvaniti, Aggelos Manolias, Maria-Aggeliki Tsatiri, Dimitra Basdani, Sokratis Zekkas

**Affiliations:** Department of Molecular Biology and Genetics, Democritus University of Thrace, 68100 Alexandroupolis, Greece

**Keywords:** Microbial genetics, Evolutionary biology, Transferases, Microbial genetics

## Abstract

Microbial NAT enzymes, which employ acyl-CoA to acylate aromatic amines and hydrazines, have been well-studied for their role in xenobiotic metabolism. Some homologues have also been linked to secondary metabolism, but this function of NAT enzymes is not as well-known. For this comparative study, we surveyed sequenced microbial genomes to update the list of formally annotated *NAT* genes, adding over 4000 new sequences (mainly bacterial, but also archaeal, fungal and protist) and portraying a broad but not universal distribution of NATs in the microbiocosmos. Localization of *NAT* sequences within microbial gene clusters was not a rare finding, and this association was evident across all main types of biosynthetic gene clusters (BGCs) implicated in secondary metabolism. Interrogation of the MIBiG database for experimentally characterized clusters with *NAT* genes further supports that secondary metabolism must be a major function for microbial NAT enzymes and should not be overlooked by researchers in the field. We also show that *NAT* sequences can be associated with bacterial plasmids potentially involved in horizontal gene transfer. Combined, our computational predictions and MIBiG literature findings reveal the extraordinary functional diversification of microbial *NAT* genes, prompting further research into their role in predicted BGCs with as yet uncharacterized function.

## Introduction

In the course of evolutionary time, microorganisms have developed immense metabolic potential and adaptability, and their capabilities have attracted scientific interest for useful biotechnological applications. Through xenobiotic metabolism, bacteria and fungi can detoxify, degrade or biotransform exogenous compounds of natural or synthetic origin, surviving and even thriving in adverse chemical environments that would be toxic to more complex organisms^1^. Microbial xenobiotic metabolism involves a plethora of enzyme activities, and arylamine *N*-acetyltransferase (NAT, E.C. 2.3.1.5) is one of them^2^. Microbial NAT enzymes catalyze the *N-*acetylation of aromatic amines, leading to detoxification of many harmful by-products of industrial activity and farming (e.g. pharmaceuticals, dyes, pesticides, etc.)^3–8^. However, they can also bioactivate procarcinogenic *N-*hydroxyarylamines via *O-*acetylation (E.C. 2.3.1.118), an activity exploited by Ames and colleagues in the popular *Salmonella* mutagenicity test^9^. The study of *Salmonella* NAT was indeed groundbreaking, in that it additionally revealed the basic structure and catalytic mechanism of the enzyme family, which employs a cysteine-histidine-aspartate (Cys-His-Asp) protease-like catalytic triad to transfer an acetyl group from donor acetyl coenzyme A (CoA) to the amino group of the acceptor aromatic amine^10,11^.

An unexpected discovery was reported for the (AMYMS)NAT3 (alias symbol RifF, GenBank ID: AFO74156.1) homologue of the actinobacterium *Amycolatopsis mediterranei* str. S699, implicating NAT not only in xenobiotic, but also in secondary metabolism. That particular homologue, which acts as an amide synthase, is encoded by a gene located at the end of the core biosynthetic gene cluster (BGC) driving production of the antibiotic rifamycin B in the actinomycete^12,13^. The reaction is atypical for a NAT enzyme, in that it employs a large polyketide chain as substrate and does not utilize acetyl-CoA. Like xenobiotic metabolism, secondary metabolism is not generally associated with vital functions of cells, but rather enhances the biological fitness of microbes as a response to environmental stress (e.g., by generating chemical weapons against competitors)^14,15^. Due to their remarkable chemical properties and variety, the products of secondary metabolism have long been exploited as a natural source of pharmaceuticals (e.g., antibiotics, anticancer agents, immunomodulating substances, etc.) and other compounds of industrial utility^16^.

A common feature of specialized microbial pathways, such as those associated with xenobiotic or secondary metabolism, is that their enzymatic components are often encoded by co-regulated genes arranged in clusters^17–19^. Activation of those gene clusters is usually triggered by specific environmental stimuli, directing resources and products of primary metabolism towards xenobiotic biotransformation or the biosynthesis of secondary metabolites. Apart from the aforementioned (AMYMS)*NAT3* (alias *rifF*) homologue of the rifamycin BGC in *A. mediterranei*, other actinobacterial *NAT* genes have also been localized in clusters associated with cholesterol degradation (specifically in slow-growing pathogenic mycobacteria) or vitamin biosynthesis (in fast-growing, free-living mycobacteria)^20–22^. Moreover, in the corn-pathogenic fungus *Fusarium verticillioides* (teleomorph *Gibberella moniliformis*), the (GIBMO)*NAT1* (alias symbol *FDB2*, GenBank ID: EU552489.1) gene, encoding the *N-*malonyltransferase that is essential for detoxification of host phytoanticipin 2-benzoxazolinone, is also part of a well-characterized gene cluster^18,23^.

Other lines of evidence suggest that certain microbial NAT homologues could play a role in secondary metabolism. For example, acyl-CoA monomers (e.g., acetyl-CoA and malonyl-CoA) derived from acetate and propionate metabolism, are employed as starter and/or extender units during the biosynthesis of polyketides^24,25^, while they are also utilized by NAT enzymes. Specifically, in addition to acetyl-CoA, NAT enzymes can utilize propionyl-CoA, butyryl-CoA and acetoacetyl-CoA as donor substrates^5,6,26–29^, while certain microbial homologues have been shown to be selective for malonyl-CoA^4,6,29^ and others can non-selectively bind various short-chain acyl-CoA compounds^6^.

The enzymatic processes of xenobiotic and secondary metabolism are believed to share an overlapping evolutionary history, while some of their key components are also encountered in fatty acid metabolism^24,30^. Although it seems likely that different NAT homologues have diverged from their ancestral forms to serve such metabolic functions in microorganisms, evidence remains sporadic and the corresponding evolutionary relationships are elusive, particularly for those NAT proteins with roles other than xenobiotic metabolism. For this comparative computational genetic study, we surveyed microbial genomes to annotate *NAT* genes, then investigating their possible localization within clusters. We also looked for possible association of *NAT* genes with bacterial plasmids, as the enzymes of xenobiotic and secondary metabolism are often encoded by genes participating in horizontal gene transfer (HGT) events involving mobile genetic elements^31^.

## Results and discussion

### Identification and annotation of microbial *NAT* genes

**Figure 1:**
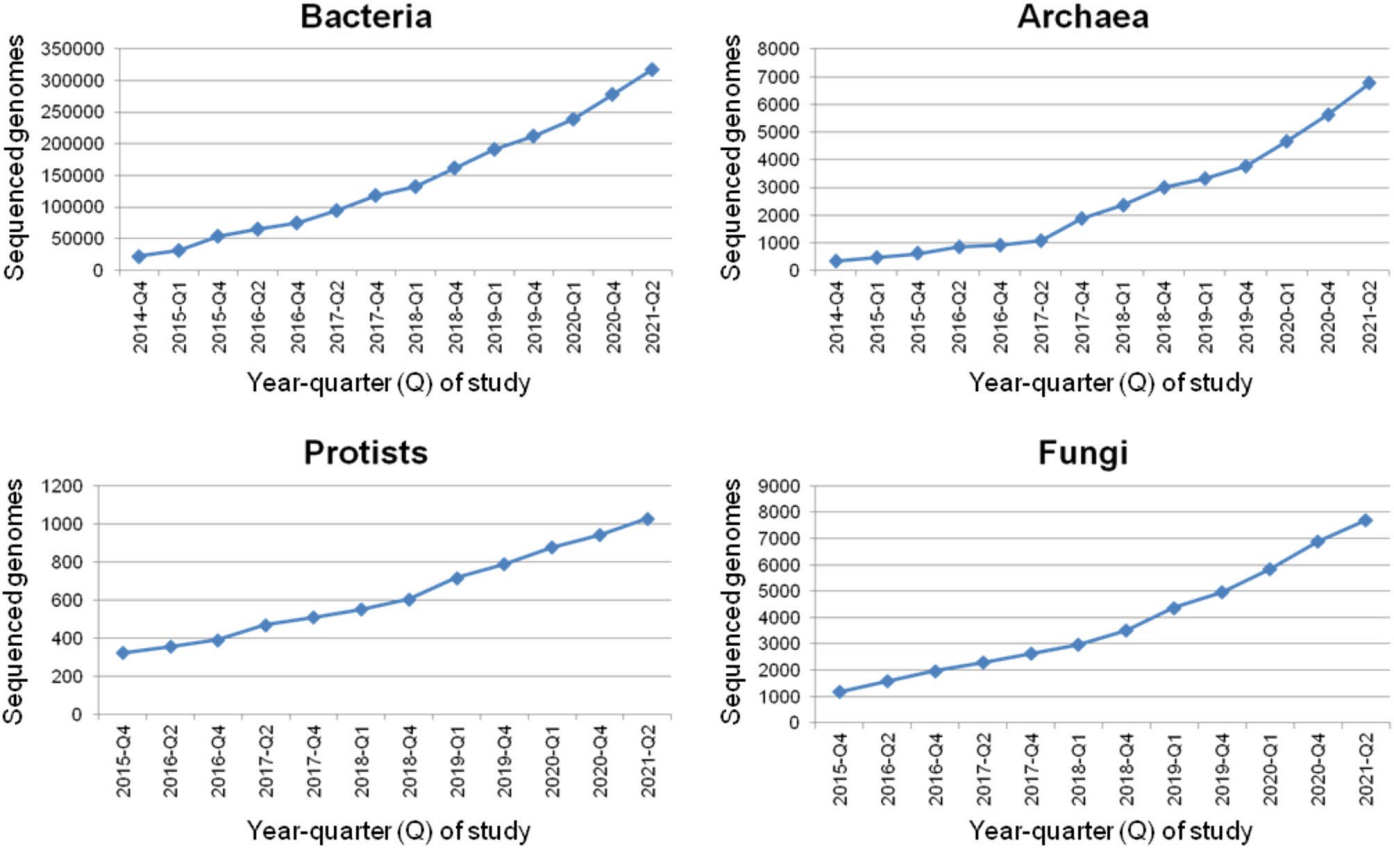
Increase in the numbers of sequenced microbial genomes deposited in the Genome database, monitored for bacteria, archaea, protists and fungi during the course of the study.

**Figure 2:**
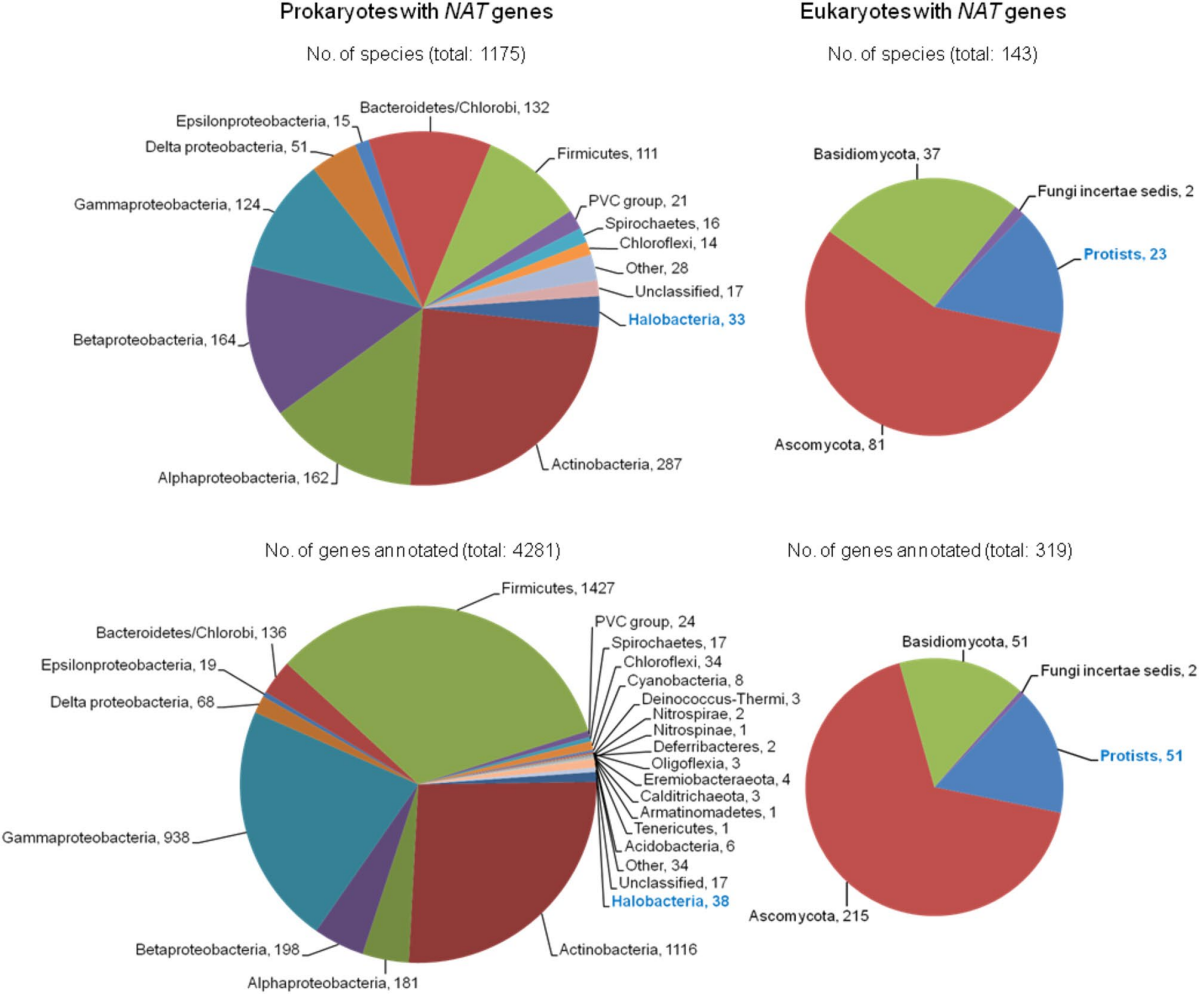
Overview of the *NAT* gene dataset compiled for the purposes of this study. The top panel depicts the main taxonomic groups represented in the dataset for prokaryotic (left) and eukaryotic (right) species. The bottom panel depicts the distribution of annotated *NAT* sequences in prokaryotes (left) and eukaryotes (right). Archaea (*Halobacteria*) and protists are indicated with blue font. The compiled *NAT* gene dataset also included 467 sequences annotated previously^32,33^.

Our previous genomic database surveys, published in 2008^32^ and 2010^33^, collectively retrieved and annotated 467 microbial *NAT* sequences (347 bacterial, 1 archaeal, 94 fungal and 25 protist), allowing the first overview of *NAT* gene distribution in the microbiocosmos. At the time of the second survey^33^, only 2,300 sequenced microbial genomes were accessible to screen, but this number has since multiplied very rapidly (Fig. 1). In view of this progress, a new survey was undertaken, to expand the earlier ones and support the analyses described later in this manuscript. The core dataset of annotated *NAT* sequences was retrieved through exhaustive database survey of approximately 34,500 prokaryotic genomes (98% bacterial, 2% archaeal; performed in 2015) and 1,400 eukaryotic genomes (68% fungal, 32% protist; performed in 2016). Additional searches were carried out later (2020–2021) to enrich the dataset, particularly with respect to previously underrepresented microbial taxa in the database. By the end of the survey, it was estimated that we had collectively covered about 324,000 prokaryotic (98% bacterial, 2% archaeal) and 8,700 eukaryotic (88% fungal, 12% protist) microbial genomes (Fig. 1). Searches were concluded for large taxonomic groups (e.g., mycobacteria, bacilli, staphylococci, burkholderias, enterobacteria, etc.) when the addition of new *NAT* genes effectively became redundant, expanding the existing set mainly with sequences from new strains of already described species. The final list (Fig. 2 and Supplementary Information S1) comprised about 4,600 annotated microbial *NAT* genes (92% bacterial, 1% archaeal, 6% fungal, 1% protist) representing 1,318 species (87% bacterial, 2.5% archaeal, 9% fungal and 1.5% protist), including the previously annotated prokaryotic and eukaryotic microbial *NAT* sequences^32,33^. The data is also available on the NAT website (http://nat.mbg.duth.gr/).

In archaea, *NAT* genes were only found in the phylum of *Euryarchaeota*, specifically in the class of *Halobacteria*. In bacteria, *NAT* genes were found in the phyla of *Acidobacteria* (classes *Blastocatellia*, *Holophagae*, *Vicinamibacteria*), *Actinobacteria*, *Armatimonadetes*, *Bacteroidetes* (FCB group), *Bdellovibrionota* (class *Oligoflexia*), *Calditrichaeota*, *Chlamydiae* (PVC group), *Chlorobi* (FCB group), *Chloroflexi*, *Cyanobacteria*, *Deferribacteres*, *Deinococcus-Thermus*, *Eremiobacteraeota*, *Firmicutes*, *Haloplasmatales/Tenericutes*, *Nitrospinae*, *Nitrospirae*, *Planctomycetes* (PVC group), *Proteobacteria* (*Alphaproteobacteria*, *Betaproteobacteria*, *Gammaproteobacteria*, *Deltaproteobacteria*, *Epsilonproteobacteria*), *Spirochaetes*, *Verrucomicrobia* (PVC group), and various unclassified bacteria. *NAT* genes were not found in the sequenced genomes from the phyla of *Aquificae*, *Chrysiogenetes*, *Coprothermobacterota*, *Dictyoglomi*, *Elusimicrobia*, *Fibrobacteres* (FCB group), *Fusobacteria*, *Krumholzibacteriota*, *Marinimicrobia*, *Synergistetes*, *Thermodesulfobacteria*, *Thermotogae* and *Caldiserica/Cryosericota* group (Fig. 2).

In protists, *NAT* genes were found in the paraphyletic clades of *Alveolata* (*Apicomplexa* and *Ciliophora*), *Amoebozoa* (*Mycetozoa/Dictyosteliida* and *Discosea/Centramoebida*), *Discoba* (*Euglenozoa* and *Heterolobosea*), and *Stramenopiles* (*Oomycetes*, *Pelagophyceae* and *Bacillariophyta*). Finally, in fungi, *NAT* genes are present in the phyla of *Ascomycota* (only *Pezizomycotina*) and *Basidiomycota*, as well as in lower fungi (*Fungi incertae sedis*) and specifically in the phyla of *Chytridiomycota* and *Zoopagomycota* (Fig. 2).

Overall, the compiled list of annotated *NAT* genes complements the previous datasets^4,29,32,33^. In prokaryotes, several new bacterial taxa with *NAT* genes were identified, while all annotated *NAT* genes of archaea belonged to halophiles, consistent with previous observation^33^. The list of *NAT* genes in eukaryotic microorganisms also expanded considerably, with new taxons added for protists, but without major changes in taxon distribution for fungi, compared with previous surveys^29,33^. On the basis of the observed sequence redundancy, it is likely that the current dataset is now effectively saturated with information and is illustrative of a broad, but not universal, distribution of *NAT* genes in microbial genomes.

### Localization of *NAT* genes in BGCs of prokaryotic microorganisms

**Figure 3:**
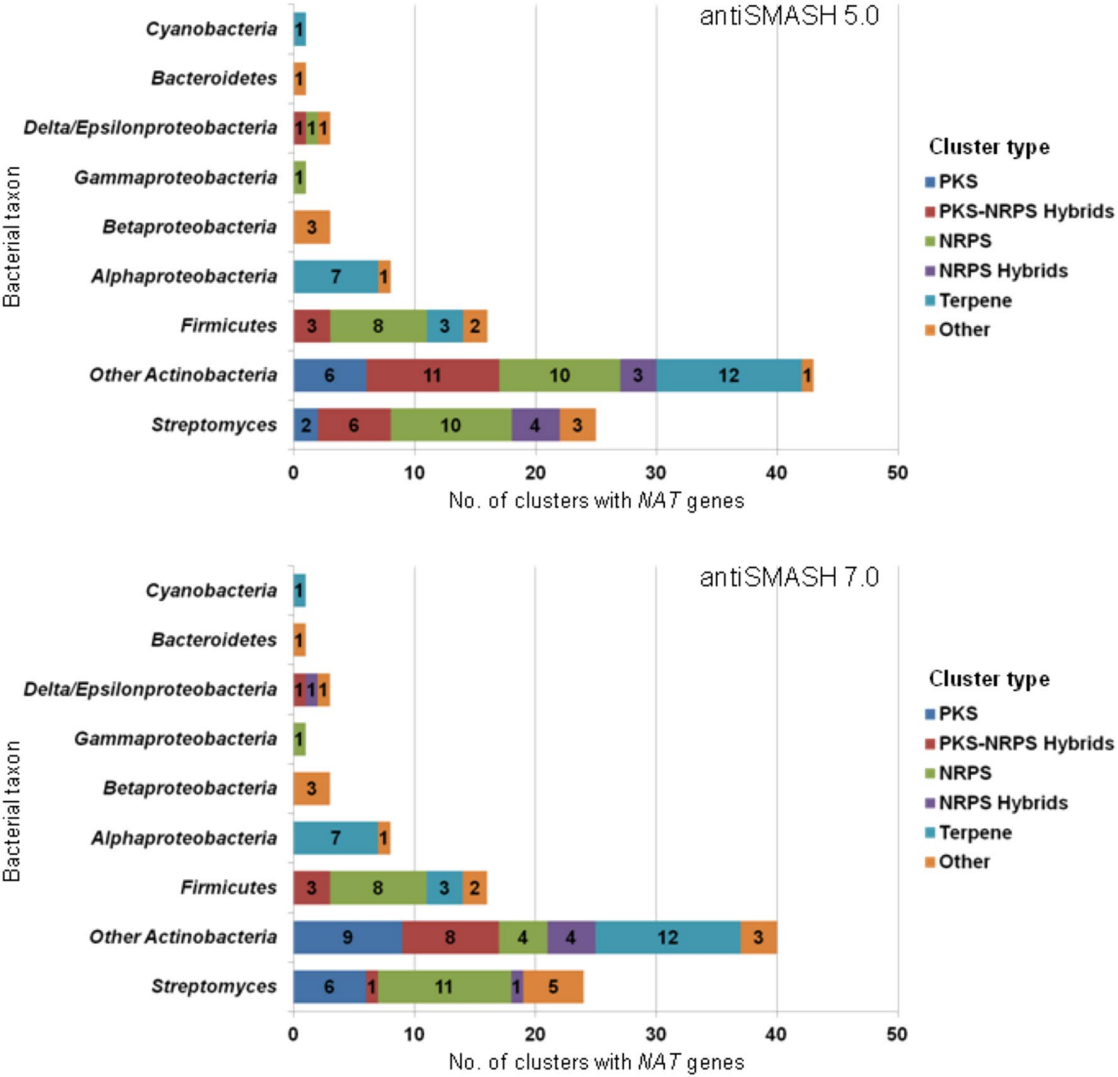
Clusters with *NAT* genes per bacterial taxon, predicted during analyses with antiSMASH software versions 5.0 (top) and 7.0 (bottom). PKS: polyketide synthase; NRPS: non-ribosomal peptide synthase. The descriptions of clusters classified as “Other” are provided in Supplementary Informations S2 and S3, for the analyses performed with antiSMASH versions 5.0 and 7.0, respectively. Note that the streptomycetes are shown separately from other actinobacteria, as they are of major importance from the point of view of secondary metabolism and the biosynthesis of natural products^35^.

The possible localization of annotated microbial *NAT* genes within genomic clusters was probed using the antibiotics and secondary metabolite analysis shell software (antiSMASH)^34^. Initially, the genomic region of 1,820 *NAT* genes was analyzed through the early antiSMASH version 3.0, and the investigation was later reiterated and expanded to include an additional 1,272 bacterial and archaeal annotated *NAT* genes, analyzed through the newer and more stringent version of antiSMASH 5.0. This screen identified 102 putative clusters bearing 103 *NAT* genes in 96 prokaryotic species, including one putative cluster with a *NAT* gene in the archaeon *Halostella salina* strain CBA1114 (Fig. 3 and Supplementary Information S2). Reanalysis of all the clusters identified with antiSMASH 5.0 was finally performed with the latest antiSMASH version 7.0, and all hits were verified, apart from four bacterial *NAT* genes which were predicted in BGCs by version 5.0, but not by version 7.0. Cluster type descriptions were also more complete with the latest version 7.0 (Fig. 3 and Supplementary Information S3). As the current version is the most accurate one, the predicted cluster coordinates and length are reported here only relative to the output of version 7.0. Refinement of BGC detection rules in the later versions provided a wider panel of predicted BGC classes, including furan, thiopeptide, linaridin, acyl-amino acid, β-lactone, arylpolyene, RiPP-like and several hybrid clusters (Fig. 3 and Supplementary Informations S2 and S3). It is, however, notable that the early (antiSMASH 3.0) version predicted several *NAT1* mycobacterial clusters which were not found by the later versions. Those clusters have been described in the literature before for *Mycobacterium bovis* BCG, but they are known to play a role in cholesterol catabolism^20^. This lack of antiSMASH 3.0 cluster prediction stringency was useful, from the point of view of our study, as it allowed comparison of an already known type of cluster across a range of different mycobacteria (Supplementary Information S4).

**Figure 4:**
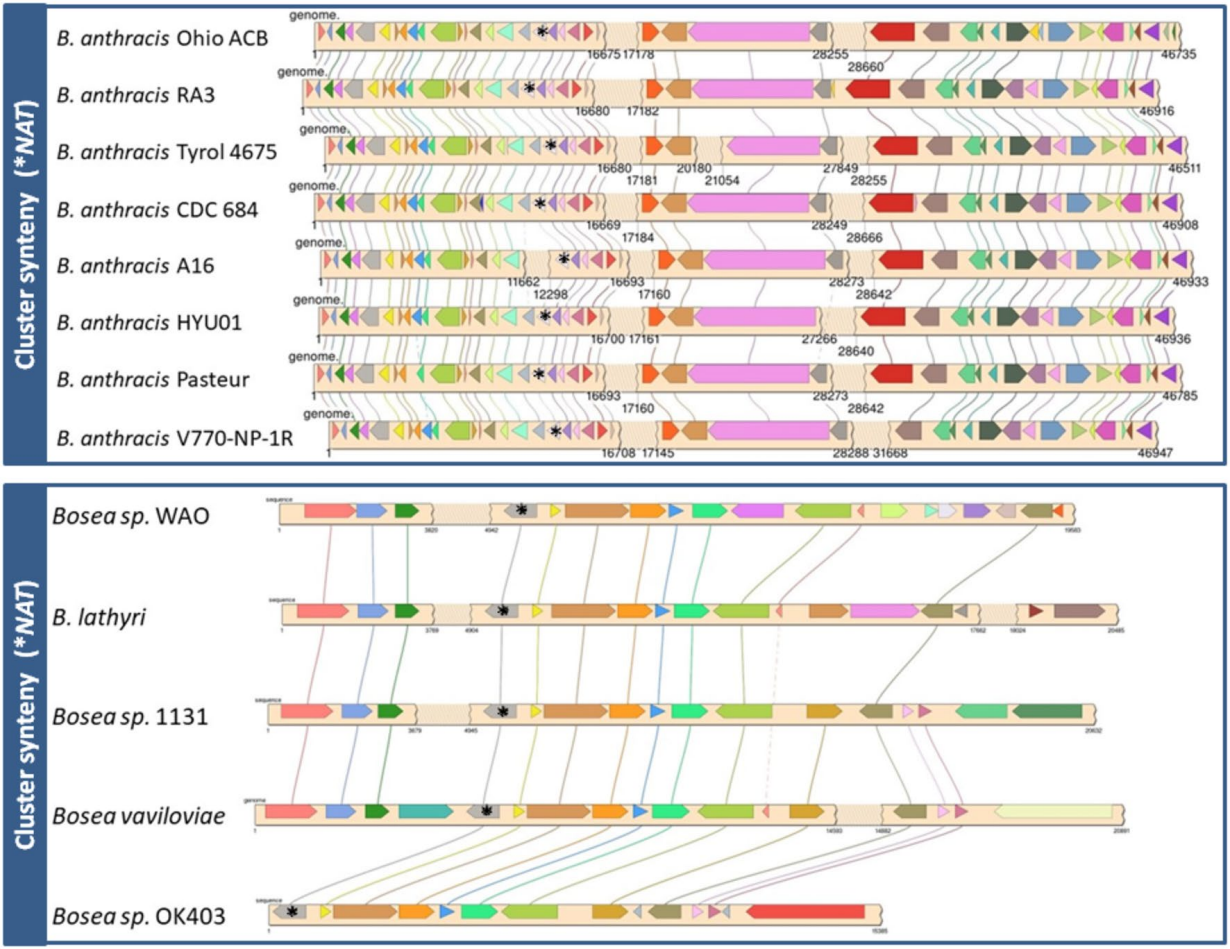
Representative illustrations of synteny between putative bacterial clusters with *NAT* genes. An example of NRPS cluster synteny between different strains of *Bacillus anthracis* (phylum *Firmicutes*) is presented in the top panel, while the bottom panel shows synteny of a terpene cluster between different species of *Bosea* (phylum *Proteobacteria*). The *NAT* gene on each cluster is indicated with an asterisk. Additional examples are provided in Supplementary Information S5.

**Figure 5:**
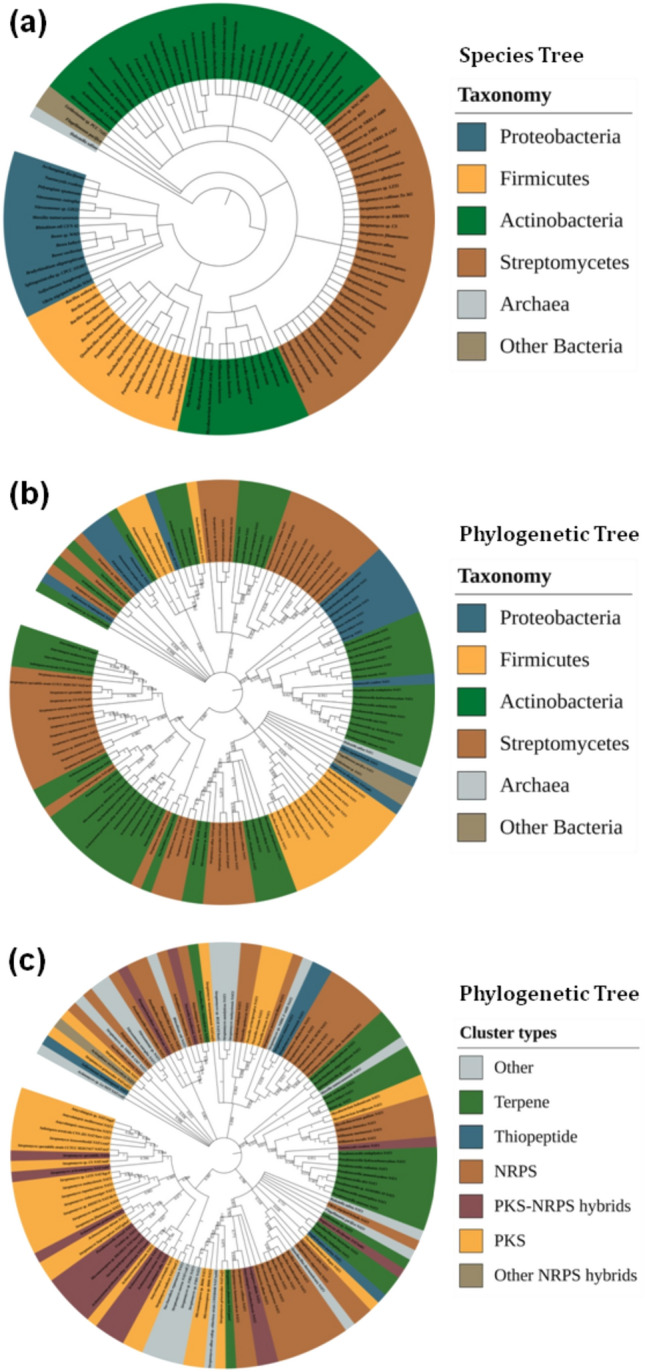
Distribution of *NAT* genes per prokaryotic taxon and type of biosynthetic gene cluster (BGC), determined during the antiSMASH 5.0 analyses (including MIBiG). The species tree (**a**) was constructed according to conventional taxonomy (NCBI Taxonomy Database common tree). The phylogenetic tree of BGC-associated NAT sequences (**b**) was constructed using the neighbour-joining method, and the leaves are coloured according to taxonomy. The same phylogenetic tree is also presented with leaves coloured according to cluster type (**c**). Note that, in a and b, the streptomycetes are shown with a different colour from other actinobacteria, as they are of major importance from the point of view of secondary metabolism and the biosynthesis of natural products^35^. The same trees are provided enlarged in Supplementary Information S6a–c, for additional clarity, alongside the corresponding trees generated with the maximum likelihood method (Supplementary Information S6d–e).

**Figure 6:**
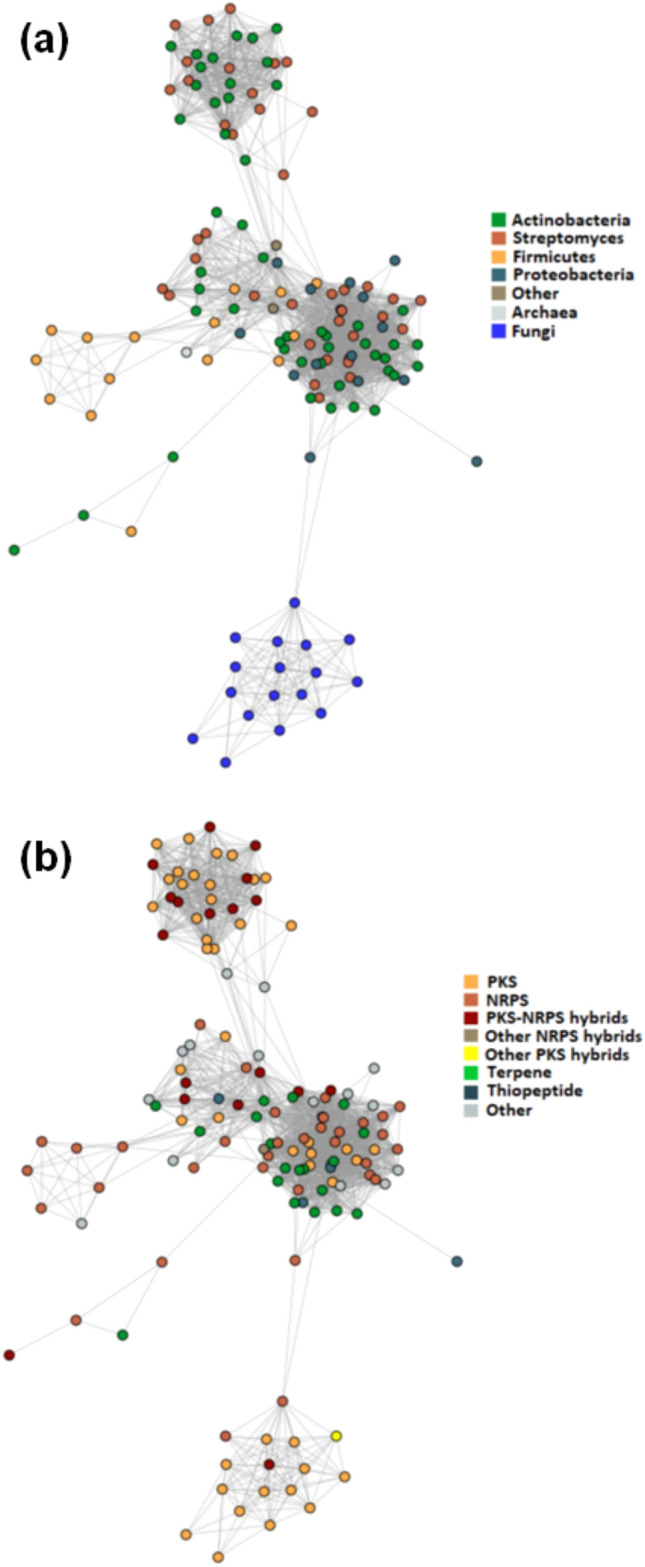
Sequence similarity network (SSN) demonstrating the relationships between different *NAT* genes found in biosynthetic gene clusters (BGCs) by antiSMASH 5.0 analyses (including MIBiG). Each node represents a BGC-associated NAT sequence and the edges connect close relatives, using an alignment score threshold of 29 (E-value = 10^−29^). The colouring of nodes is either according to taxonomic group (**a**) or according to cluster type (**b**). Note that, in a, the streptomycetes are shown with a different colour from other actinobacteria, as they are of major importance from the point of view of secondary metabolism and the biosynthesis of natural products^35^.

In view of the known association of (AMYMS)*NAT3* (*rifF*) gene with the BGC of rifamycin in *A. mediterranei*^12,13^, it was expected that antiSMASH would detect *NAT* genes only in conserved actinobacterial polyketide synthase (PKS) clusters responsible for the biosynthesis of ansamycin antibiotics like rifamycin. Surprisingly, this was not the case, as the software predicted different *NAT* genes within a spectrum of BGC types (Fig. 3 and Supplementary Informations S2 and S3), implying that the enzymatic function of NAT proteins in secondary metabolism is unlikely to be restricted merely to the amide synthase activity reported for (AMYMS)NAT3 (RifF). The diversity in the gene content and organization of BGCs harbouring *NAT* homologues was indeed remarkable, with synteny between clusters observed just for different strains of the same species and only partially between closely related species of the same genus (see examples in Fig. 4 and Supplementary Information S5). It was also apparent that *NAT* genes are not associated with BGCs restricted to a specific taxonomic group of bacteria, as phylogenetic analyses demonstrated that the distribution of BGC-associated NAT homologues is intermixed, with low basal resolution across different taxa. More specifically, in the phylogenetic trees of Fig. 5 and Supplementary Information S6, the distribution of BGC-associated NAT sequences in different clades is neither according to taxonomy, nor according to BGC type. In contrast, BGC-associated NATs illustrate a mosaic distribution pattern that spans different bacterial groups, suggesting widespread HGT events, not just at the level of individual genes (as has been reported before^4,33^), but also at the level of whole BGCs. For example, the NATs of terpene BGCs appear to cluster together in the phylogenetic tree, although some of them belong to alphaproteobacteria and some to actinobacteria (Fig. 5b,c and Supplementary Information S6b–e). The same mosaic distribution of BGC-associated NATs is also observed in the sequence similarity networks (SSNs) of Fig. 6, showing a highly intermixed core group (whether it is viewed from the standpoint of taxonomy or of BGC type), connected with two more specialized groups of homologues. The first group contains certain *Firmicutes* NATs associated with non-ribosomal peptide synthase (NRPS) clusters, while the second group comprises the actinobacterial NATs associated with PKS or PKS-NRPS hybrid clusters that are responsible for the biosynthesis of ansamycins (Fig. 6). In those last BGCs, the *NAT* genes are likely to be orthologous to *rifF*.

As the actinobacteria, and particularly the streptomycetes, represent the richest source of bacterial secondary metabolites^36^, it is perhaps unsurprising that the majority (66%) of BGCs with *NAT* genes were identified to belong to this particular taxonomic group (Fig. 3). Moreover, about 60% of those actinobacterial clusters were predicted to belong to the BGC types of NRPS, PKS, PKS-NRPS hybrid or NRPS hybrid. In those biosynthetic pathways, scaffold assembly is regarded to proceed through successive rounds of chain elongation, using acyl-CoA molecules (in PKS clusters) or amino acids (in NRPS clusters) as extension units^24,25^. The ability of NAT enzymes to accommodate aromatic amines and short-chain acyl-CoA molecules in their active site may partially explain the recruitment of microbial *NAT* genes by the NRPS/PKS system. Moreover, although those assembly lines are typically terminated by thioesterases, the example of the (AMYMS)NAT3 (RifF) amide synthase demonstrates that other homologous NATs could also serve the release of fully assembled scaffolds from the biosynthetic machinery^37^. It is also possible that NAT enzymes may be implicated in chemical modification of the peptide or polyketide core structure, contributing to chemical diversification of the end product.

About 17% of identified BGCs with *NAT* genes were found in *Firmicutes*, mainly bacilli. Most of those BGCs were of the NRPS type and were associated with NAT3 isoforms, such as those of *Bacillus anthracis* and *Bacillus cereus* which have been expressed in recombinant form and tested for catalytic activity against arylamines^38–40^. Although active, the (BACCE)NAT3 isoenzyme of *B. cereus* deviates from other functionally characterized NATs in that it has a catalytic triad with Glu instead of Asp^39^. In contrast, although endogenously expressed, the (BACAN)NAT3 of *B. anthracis* is substantially shorter and apparently non-functional as *N-*acetyltransferase, due to its gene being compromised by a frameshift mutation^32^. It is tempting to speculate whether those unusual features of NAT3 in bacilli could serve some specific function in the associated NRPS cluster, especially since studies have shown that truncation of the C-terminus may convert NATs into acetyl-CoA hydrolases^41,42^.

Unlike *Actinobacteria* and *Firmicutes*, in *Proteobacteria* only a few *NAT* genes were predicted within BGCs. In alphaproteobacteria, those are involved in the biosynthesis of terpenes which differs substantially from that of polyketides and non-ribosomal peptides. Therefore, the NAT enzymes participating in those pathways could differentiate functionally. For instance, as the core hydrocarbon skeleton of terpenes is modified, e.g. by addition of amino acids or fatty acids^43^, NAT could act as acyltransferase or as modulator of acyl-CoA availability, like it has been suggested before for mycobacteria^44^. It is also of note that two *NAT* genes of *Bradyrhizobium oligotrophicum* are localized within the same terpene BGC.

In betaproteobacteria, all three BGCs with *NAT* genes were predicted to direct the synthesis of acyl-amino acids. Those NAT enzymes could act as acyltransferases, and recent work has demonstrated human NAT2 to be capable of employing not just aromatic, but also aliphatic amines as substrates^45^. The remaining BGCs with *NAT* genes in gamma, delta and epsilonproteobacteria were of various types and only sporadic, most likely the outcome of HGT from other bacterial groups. The same is also probable for the β-lactone BGC found in the archaeon. In conclusion, it is likely that once associated with secondary metabolism, *NAT* genes had broad opportunity to diverge from their archetypal function to serve a range of biosynthetic processes.

### Localization of *NAT* genes in BGCs of eukaryotic microorganisms

As BGCs are also known to drive secondary metabolism in fungi^15,17^, the 268 *NAT* genes annotated during the genomic survey described above (Supplementary Information S1) and previously^33^ were investigated as to their possible localization within clusters. The procedure was the same as for prokaryotes, and the results of the analyses with antiSMASH versions 3.0, 5.0 and 7.0 were compared. As in prokaryotes, the earliest less stringent version 3.0 localized certain functionally investigated *NAT* genes^6^ within clusters, namely, *NAT1* (encoding for *N-*malonyltransferase) and *NAT3* (encoding for *N-*acetyltransferase) found in *Fusarium graminearum* str. PH-1 and *F. oxysporum f.sp. lycopersici* str. 4287, as well as the (GIBMO)*NAT3* of *F. verticillioides* str. 7600 and the (ASPFN)*NAT3* of *A. flavus* str. NRRL 3357. The *NAT4* homologue^6^ of various *F. oxysporum* types was also predicted to be associated with BGCs.

When the analysis was repeated with the later antiSMASH version 5.0, the number of recovered hits was considerably smaller, but much more accurately annotated (Supplementary Information S7). All 16 fungal BGCs, identified to harbour *NAT* genes, belonged to filamentous ascomycetes. Of those, 13 belonged to *Eurotiomycetes* and they were predicted to function as PKS or PKS hybrid clusters. Only 3 BGCs with *NAT* were predicted in *Sordariomycetes*, and these were mainly of the NRPS type (Supplementary Information S7). Reanalysis of those results with the latest version of antiSMASH 7.0 verified the hits, also updating matches with experimentally characterized BGCs like the PKS cluster for 8-methyldiaporthin of *A. flavus* str. RIB40^46^ (Table 1 and Supplementary Information S8). As expected, in the SSN of Fig. 6, the fungal and bacterial sequences were separate, consistent with the monophyletic origin of fungal *NAT* genes^33^.

**Table 1:** Fungal *NAT* genes predicted to localize within biosynthetic gene clusters (BGCs) by antiSMASH version 7.0.

Organism scientific name	Fungal *NAT* genes within BGCs			Functionally characterized BGCs similar to those predicted (MIBiG database)		
	*NAT* gene	Contig accession number: cluster coordinates	BGC type^a^	MIBiG BGC ID	BGC product	% of genes providing BLAST hits
*Aspergillus bombycis* strain NRRL26010	*NAT2*	LYCR01000072.1: 10156..56625	T1PKS	BGC0002236	8-Methyldiaporthin	100
*Aspergillus flavus* AF70	*NAT3*	JZDT01000919.1: 198365..244830	T1PKS	BGC0002236	8-Methyldiaporthin	100
*Aspergillus kawachii* IFO 4308	*NAT3*	DF126457.1: 1..29919	T1PKS	N/A		
*Aspergillus niger* An76	*NAT4*	BCMY01000002.1: 1137426..1186595	T1PKS	N/A		
*Aspergillus oryzae* RIB40	*NAT3*	NW_001884682.1: 92784..138782	T1PKS	BGC0002236	8-Methyldiaporthin	100
*Aspergillus oryzae* 100-8	*NAT3*	AMCJ01000103: 878641..925106	T1PKS	BGC0002236	8-Methyldiaporthin	100
*Aspergillus parasiticus* SU-1	*NAT3*	JZEE01000186.1: 133999..180453	T1PKS	BGC0002236	8-Methyldiaporthin	100
*Aspergillus piperis* CBS 112811	*NAT1*	NW_020291594.1: 251331..299010	T1PKS	N/A		
*Aspergillus pseudotamarii* CBS 117625	*NAT1*	NW_022475042.1: 11899..60965	T1PKS	N/A		
*Aspergillus udagawae* IFM 46973	*NAT3*	BBXM01000084.1: 68242..116571	T1PKS	BGC0002525	Fusarubin, 1233A, 1233B, NG-391, lucilactaene	28
*Penicillium expansum* MD-8	*NAT1*	NW_015971216.1: 72103..163635	T1PKS, Terpene	BGC0001338	Citrinin	56
*Penicillium nordicum* DAOMC 185683	*NAT1*	LHQQ01000075.1: 1..52004	T1PKS	N/A		
*Penicillium polonicum* IBT 4502	*NAT1*	MDYM01000009.1: 157770..250263	NRPS, T1PKS, Terpene	BGC0002710	Metachelin C, A, A-CE, B, dimerumic acid 11-mannoside, dimerumic acid	50
*Acremonium chrysogenum* ATCC 11550	*NAT2*	JPKY01000133: 1..34441	Indole-T1PKS	N/A		
*Myceliophthora thermophila* ATCC 42464	*NAT1*	CP003002.1: 2483801..2485324	NRPS	BGC0002158	Tenuazonic acid	50
*Verticillium albo-atrum* VaMs.102	*NAT1*	DS985223.1: 1005871..1038902	NRPS-like	N/A		

See Supplementary Information S8 for complete record.

^a^BGC types: T1PKS, Type I polyketide synthase; NRPS, Non-ribosomal peptide synthase.

Finally, no hits were provided by antiSMASH analyses of 51 annotated *NAT* sequences from protists, reported here (Supplementary Information S1) and in our previous study^33^. The only possible exception was (DICDI)*NAT4* of *Dictyostelium discoideum* str. AX4 which could reside in a BGC. In addition to gene annotations provided by the GenBank, in the future it may be useful to also try different eukaryotic gene-calling algorithms, like Augustus^47^, to investigate the genomic context of *NAT* loci in fungi and protists.

### Localization of *NAT* genes in bacterial plasmids

Although the Genome database reported almost 30,000 sequenced plasmids at the time of the study, those sequences were not accessible by BLAST via the NCBI and so instead we looked for them via the specialized PLSDB database^48^. A total of 92 bacterial plasmids were identified to carry 117 *NAT* genes in several actinobacteria, alphaproteobacteria, betaproteobacteria, gammaproteobacteria and bacilli (Table 2 and Supplementary Information S9). Those plasmids were either circular or linear, and their size varied from about 30.3 Kb (plasmid pYGD30 of *Bacillus thuringiensis* strain YGd22-03) to 2.8 Mb (plasmid of *Cupriavidus campinensis* strain MJ1). It is noteworthy that several of the identified plasmids carry more than one *NAT* gene, particularly in the bacilli which often display multiple *NAT* open reading frames (ORFs) in their plasmids, similarly to their genomic sequence. Those included ORFs with frameshift mutations, as has been reported previously for the genomic *NAT3* homologues of certain bacilli^32^.

**Table 2:** Overview of bacterial plasmids carrying *NAT* genes.

Taxonomic group	Genus	Number of species/strains	Number of plasmids	Number of *NAT* genes
*Actinobacteria*	*Streptomyces*	6	7	8
	*Tsukamurella*	1	1	1
*Alphaproteobacteria*	*Ensifer*	2	2	2
	*Rhizobium*	3	3	3
	*Sinorhizobium*	2	2	2
*Betaproteobacteria*	*Caballeronia*	2	2	2
	*Cupriavidus*	3	3	4
	*Mycetohabitans*	1	1	1
*Gammaproteobacteria*	*Klebsiella*	23	23	23
	*Erwinia*	1	1	1
	*Pantoea*	3	3	3
	*Vibrio*	4	4	4
*Firmicutes*	*Bacillus*	34	37	59
	*Brevibacillus*	1	1	1
	*Paenibacillus*	2	2	3

See Supplementary Information S9 for complete record.

**Figure 7:**
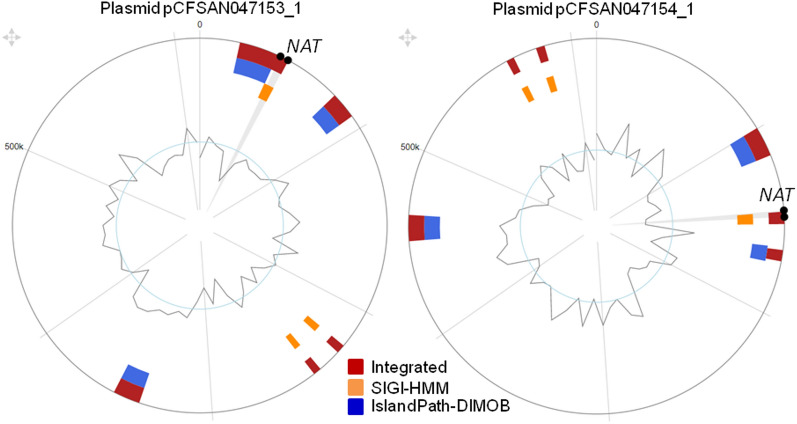
Plasmid genomic islands harbouring *NAT* genes. The genomic islands of two plasmids carried by strains CFSAN047153 and CFSAN047154 of the gammaproteobacterium *Pantoea agglomerans*, were predicted by IslandViewer algorithms SIGI-HMM and IslandPath-DIMOB. The outer circle is the plasmid and the graphical illustration of the inner circle is the GC content of the corresponding sequence, as deviation from the expected GC content may be indicative of heterologous portions originated through horizontal gene transfer (HGT). The *NAT* gene is located between the black dots, within a low-GC genomic island.

All plasmid-associated *NAT* genes were subsequently screened by antiSMASH 6.0 for possible localization within BGCs, and this was confirmed for five of them (Table 3). Finally, all identified plasmids were screened for the presence of genomic islands, which are indicative of exchanges between plasmid and chromosomal DNA in bacteria^49^. Such genomic islands were identified to harbour *NAT* genes in five different plasmids, but only the plasmids of the gammaproteobacterium *Pantoea agglomerans* were found to carry intact ORFs without frameshift mutations (Fig. 7).

**Table 3:** Overview of bacterial plasmids carrying *NAT* genes within biosynthetic gene clusters (BGCs).

Organism scientific name	Plasmid name	*NAT* gene	*NAT* gene locus tag Protein ID	BGC type (MIBiG)^a^	Compound (MIBiG)
*Streptomyces parvulus* strain 2297	pSPA1	*NAT1*	Spa2297_RS32575 WP_079163890.1	NRPS NRPS-like T1PKS Betalactone Butyrolatone Other	Polyoxypeptin
*Streptomyces* sp. Mg1	pSMg1-3	*NAT1*	M444_RS37885/ WP_047961327.1	NRPS-like T1PKS Arylpolyene Butyrolactone Other	Neocarzinostatin
*Streptomyces reticuli* TUE45	Plasmid: II	*NAT1*	TUE45_pSRTUE45c_0202 CUW32834.1	T1PKS T3PKS Aminocoumarin Lassopeptide Nucleoside Terpene	Rubradirin
*Bacillus mycoides* strain Gnyt1	Unnamed1	*NAT1*	B7492_RS30070 WP_061676092.1	CDPS	–
*Paenibacillus cellulositrophicus* strain KACC 16577	Unnamed1	*NAT1*	GCU45_RS30450 WP_152403617.1	NRPS-like	–

^a^BGC types: T1PKS, Type I polyketide synthase; T3PKS, Type 3 polyketide synthase; NRPS, Non-ribosomal peptide synthase.

Genes like *NAT*, implicated in xenobiotic and secondary metabolism, are often encountered in plasmids and are exchanged between bacterial cells enhancing adaptability to adverse environmental conditions. Moreover, BGCs introduced from plasmids can enhance the biosynthetic capabilities of hosts^50,51^. In that respect, plasmids with *NAT* genes may enhance the ability of bacterial cells to detoxify potentially harmful xenobiotics in their environment. Moreover, *NAT* genes carried by plasmids were also found to be associated with BGCs. For example, in plasmid II of *Streptomyces reticuli* str. TUE45, the *NAT* gene is located within a predicted BGC for the ansamycin antibiotic rubradirin^52^, where it is predicted to act as an amide synthase similar to (AMYMS)NAT3 (RifF). Furthermore, BLAST search of the *NAT* sequence found in the genomic island of the *P. agglomerans* plasmid, demonstrates a good match with chromosomal gene *Pnp2A* that is homologous to *NAT* and is part of a six-gene BGC responsible for antibiotic biosynthesis^53^.

### Interrogation of the MIBiG database for *NAT* genes associated with experimentally characterized BGCs

A significant aim of the present work was to assess the amount of information available in the literature, regarding the genomic and functional links of microbial *NAT* genes with secondary metabolism. For decades, this information has been increasing in volume, but has effectively stayed under the radar of scientists dedicated to NAT research, because of a gap in gene nomenclature. Specifically, it is common practice for researchers characterizing new BGCs to name genes after the cluster they are located in and according to their genomic order. For example, (AMYMS)*NAT3* of *A. mediterranei* was identified to be the sixth gene (*F*) on the core BGC for rifamycin (*rif*), so it was named *rifF*. Moreover, the protein product of this gene was described based on function (amide synthase), rather than homology to other NAT enzymes^12,13,37,54^. Consequently, using the keywords “NAT” or “arylamine *N-*acetyltransferase” to search PubMed cannot readily pick up relevant literature. Hence, with the exception of *rifF*^55^, studies directly connecting NATs with their BGC-associated homologues are effectively lacking and microbial NATs have been functionally investigated as xenobiotic metabolizing enzymes.

Modern databases provide access to the literature, enabling search with a gene/protein sequence instead of keywords. One such database is MIBiG (minimum information about a biosynthetic gene cluster)^56^, used in this study as part of the antiSMASH searches described above. In addition, the whole MIBiG sequence repository was downloaded and subjected to BLAST search with NAT sequences as query. This database is dedicated to depositing information about experimentally characterized BGCs and their chemical products, thus, any NAT sequences recovered would be expected to be part of an already characterized gene cluster.

Indeed, the interrogation of MIBiG database identified several characterized *NAT* homologues within bacterial BGCs, for which literature was already available (Table 4). Apart from *A. mediterranei*, the marine actinomycete *Salinispora arenicola* has been demonstrated to possess a rifamycin BGC carrying a *NAT/rifF* orthologue^57,58^. Other BGCs responsible for the production of ansamycin secondary metabolites have been experimentally characterized in actinomycetes and, based on sequence comparison and chemical analogy of the synthesized product, the corresponding NAT homologues are proposed to have an amide synthase function similar to RifF. Ansamycins are medicinally important compounds characterized by an aliphatic (ansa) chain linked to non-adjacent positions of a benzene- or naphthalene-based chromophore^59,60^. Benzenic ansamycins (e.g. geldanamycin, macbecin and ansamitocin in Table 4) are known for their cytotoxic action against eukaryotic cells, while naphthalene-based ansamycins (e.g. rifamycin and its congeners, rubradirin, streptovaricin and naphthomycin in Table 4) exhibit mainly antimicrobial activity. Despite the structural variation of the produced metabolites, biosynthetic pathways of ansamycins share crucial similarities, reflected in the organization of the corresponding BGCs. The main part of those clusters is typically occupied by genes encoding a PKS. Directly downstream there is usually a *NAT* gene, followed by genes responsible for 3-amino-5-hydroxybenzoic acid (3,5-AHBA) biosynthesis, which serves as the universal precursor for ansamycin polyketides synthesized by the PKS machinery^59,60^. The assembled linear product then serves as substrate for the NAT amide synthase, which links the carboxyl to the arylamine end of the polyketide chain, simulating the typical donor–acceptor substrate reaction of NAT enzymes. Consistent with the known NAT catalytic mechanism^61^, the first step for ansamycin macrolactamization is likely to involve covalent attachment of the polyketide aliphatic end to catalytic Cys^62^. Completion of the reaction requires that the two ends of the polyketide substrate come into close proximity, indicating that the catalytic pocket is large enough to accommodate such a bulky substrate. The modelled structure of RifF has a loop, instead of the typical helix, between domains II and III, potentially rendering entry to the active site less restricted relative to other NATs^55^.

**Table 4:** List of *NAT* genes located within experimentally characterized biosynthetic gene clusters (BGCs), identified via interrogation of the MIBiG database.

Organism scientific name	*NAT* gene	NAT protein ID (NCBI)	Proposed NAT function^a^	BGC type	BGC ID (MIBiG)	BGC product	BGC product activity^b^	References
*Amycolatopsis mediterranei* strain S699	*NAT3* *rifF*	AAC01715.1	Amide synthase (polyketide cyclization)	PKS	BGC0000136	Rifamycin	Antimicrobial	^12,13,63^
*Salinispora arenicola* strain CNS-205	*NAT* *sare_1251*	ABV97156.1	Amide synthase (polyketide cyclization)	PKS	BGC0000137	Rifamycin	Antimicrobial	^57,58^
*Streptomyces* sp. strain CS	*NAT* *natF*	ADM46361.1	Amide synthase (polyketide cyclization)	PKS	BGC0000106	Naphthomycin A	Antimicrobial, antitumor	^64^
*Streptomyces* sp. strain HKI0576 (*Streptomyces* sp. strain W112)	*NAT* *divN*	CCP20052.1	Amide synthase (polyketide cyclization)	PKS	BGC0001119	Divergolide A-D	Antimicrobial, antitumor	^65,66^
*Streptomyces* sp. strain LZ35	*NAT* *hgcF*	AFV30252.1	Amide synthase (polyketide cyclization)	PKS	BGC0000075	Hygrocin A,B	Antimicrobial, antitumor	^66^
*Streptomyces leeuwenhoekii* strain C34	*NAT2* *cxmF*	CQR60492.1	Amide synthase (polyketide cyclization)	PKS	BGC0001287	Chaxamycin A-D	Antimicrobial, antitumor	^67^
*Amycolatopsis* sp. strain Hca4	*NAT* *rmpF*	AWH12663.1	Amide synthase (polyketide cyclization)	PKS	BGC0001759	Rifamorpholine A-E	Antimicrobial	^68^
*Streptomyces spectabilis* strain CCTCC M2017417	*stvF*	ASZ00152.1	Amide synthase (polyketide cyclization)	PKS	BGC0001785	Streptovaricin	Antimicrobial	^69^
*Amycolatopsis vancoresmycina* strain NRRL B-24208	*NAT5* *kngF*	WP_004559807.1	Amide synthase (polyketide cyclization)	PKS	BGC0002009	Kanglemycin A,V1,V2	Antimicrobial	^70^
*Streptomyces achromogenes* subsp. *rubradiris* strain NRRL 3061	*rubF*	CAI94702.1	Amide synthase (polyketide cyclization)	PKS	BGC0000141	Rubradirin	Antibiotic	^52,71^
*Actinosynnema pretiosum* subsp. *auranticum* strain ATCC 31565	*asm9*	AAM54087.1	Amide synthase (polyketide cyclization)	PKS	BGC0000020	Ansamitocin P-3	Antitumor	^72^
*Actinosynnema pretiosum* subsp. *pretiosum* strain ATCC 31280	*NAT3* *ansa11*	AQZ37096.1	(–) (*asm9* of BGC0000020, 97% identity/ 99.6% coverage)	PKS	BGC0001511	Ansamitocin P-3	Antitumor	^73^
	*NAT2* *mbcF*	ACF35448.1	Amide synthase (polyketide cyclization)	PKS	BGC0000090	Macbecin	Antitumor	^74^
*Streptomyces hygroscopicus* strain NRRL 3602	*gdmF*	AAO06919.1	Amide synthase (polyketide cyclization)	PKS	BGC0000066	Geldanamycin	Antitumor	^75^
*Streptomyces hygroscopicus* subsp. *duamyceticus* strain JCM4427	*gelD*	ABB86411.1	(–) (*gdmF* of BGC0000066, 100% identity/ 100% coverage)	PKS	BGC0000067	Geldanamycin	Antitumor	^76^
*Streptomyces hygroscopicus* 17997	*gdmF*	ABI93780.1	Amide synthase (polyketide cyclization)	PKS	BGC0000068	Geldanamycin	Antitumor	^77^
*Micromonospora* sp. strain HK160111	*NAT* *mas10*	ATY46593.1	Amide synthase (polyketide cyclization)	PKS	BGC0001666	Microansamycins A-I	Unknown	^78^
*Amycolatopsis alba* strain DSM 44262	*NAT1* *asc9*	WP_020636846.1	Amide synthase (polyketide cyclization)	PKS	BGC0002011	Ansacarbamitocin A	Antibiotic	^79^
*Streptomyces nodosus* subsp. *asukaensis* strain ATCC 29757	*asuC2*	ADI58636.1	*N*-acyltransferase	T2PKS	BGC0000187	Asukamycin	Antimicrobial, antitumor	^80^
*Streptomyces aureus* SOK1/5-04	*NAT* *colC2*	AIL50169.1	*N*-acyltransferase	T2PKS	BGC0000213	Colabomycin E	Anti-inflammatory, antibiotic	^81^
*Streptomyces platensis* MA7327	*ptmC*	ACO31290.1	Arylamine *N*-acyltransferase (substrates: ADHBA and platensicyl-CoA or platencinyl-CoA)	Terpene	BGC0001140	Platensimycin, platencin	Antibiotic	^82–84^
*Streptomyces platensis* MA7339	*ptnC*	ADD82996.1	Arylamine *N*-acyltransferase (substrates: ADHBA and platencinyl-CoA)	Terpene	BGC0001156	Platencin	Antibiotic	^82–84^
*Streptomyces albus* subsp. *chlorinus* strain LW030448 (NRRL B-24108)	*nybK*	AYV61412.1	Arylamine* N*-acyltransferase (substrates: acetoacetyl-CoA and 2,6-diaminophenol)	Other	BGC0001965	Nybomycin	Antibiotic	^85^
*Streptomyces* sp. F001	*NAT2* *daqS*	RZB16698.1	Arylamine *N*-acyltransferase (substrates: 2,6-DAHQ and β-ketoacyl-CoA)	Other	BGC0001850	Diazaquinomycin A,E,F,G	Antibiotic, antitumor	^86^
	*NAT3* *daqT*	RZB16697.1						
*Micromonospora* sp. B006	*NAT1* *daqS*	AXO35214.1	Arylamine* N*-acyltransferase (substrates: 2,6-DAHQ and β-ketoacyl-CoA)	Other	BGC0001848	Diazaquinomycin H,J	Antibiotic	^86^
	*NAT2* *daqT*	AXO35215.1						
*Actinomyces* sp. Lu 9419	*NAT* *cetD*	ABL74384.1	Aminocyclitol *N*-acetyltransferase	Cyclitol	BGC0000283	Cetoniacytone A	Antitumor	^87,88^
*Streptomyces* sp. NRRL B-1347	*NAT2* *gilW*	WP_030684641.1	Putative *N*-acetyltransferase	Other	BGC0001607	Gilvusmycin	Antibiotic, antitumor	^89^
*Archangium disciforme* *Angiococcus disciformis* An d48	*NAT* *tubG*	CAF05656.1	*O*-acyltransferase	NRPS–PKS	BGC0001053	Tubulysin A	Cytotoxic, anticancer	^90^
*Streptomyces* sp. RI18	*NAT* *bezG*	BBC27534.1	*O*-acetyltransferase (substrates: PHABA and acetyl-CoA), essential for the formation of the bicyclic scaffold found in the final product	Other	BGC0001529	Benzastatin derivatives	Antioxidant	^91^
*Streptomyces murayamaensis* sp. nov. Hata et Ohtani	*NAT* *orf3*	AAO65324.1	(–) (*bezG* of BGC0001529, 47.0% identity/97.8% coverage)	PKS	BGC0000236	Kinamycin	Antimicrobial, antitumor	^92^
*Streptomyces griseoruber* strain Sgr29	*NAT* *orf2*	AQW35032.1	(–) (*nybK* of BGC0001965, 40% identity/100.4% coverage)	PKS	BGC0001675	Murayaquinone	Antibiotic	^93^
	*NAT* *orf23*	AQW35053.1	(–) (*orf3* of BGC0000236, 43% identity/92.5% coverage)					

PKS, Polyketide synthase; T2PKS, Type 2 polyketide synthase; NRPS, Non-ribosomal peptide synthase; ADHBA, 3-amino-2,4-dihydroxybenzoic acid; 2,6-DAHQ, 2,6-diaminohydroquinone; PHABA, *p*-hydroxyaminobenzoic acid.

^a^The proposed function of each NAT homologue was extracted from the cited paper. For NAT homologues lacking functional description (–), the most similar MIBiG protein entry is reported.

^b^Bioactivity of the biosynthetic product is according to the cited paper or the PubChem database (https://pubchem.ncbi.nlm.nih.gov/).

Several of the *NAT* homologues of Table 4 are involved in biosynthetic pathways that link substrate molecules via an amide bond. For example, *asuC2* of *Streptomyces nodosus* and *colC2* of *Streptomyces aureus* encode NAT homologues that are proposed to participate in the biosynthesis of the pokyketides asukamycin and colabomycin E, respectively^80,81^. The metabolic phenotype of an *asuC2* knockout strain indicates that NAT acts as the amide synthase performing the attachment of the upper polyketide chain to the amino group of 3-amino-4-hydroxybenzoic acid (3,4-AHBA)^80^. Similarly to its isomer 3,5-AHBA, this compound is a precursor in the biosynthesis of secondary metabolites, e.g. the terpene pigment grixazone produced by *Streptomyces griseus.* Although the *NAT* homologue of this actinomycete is not part of the grixazone BGC, the encoded protein can *Ν*-acetylate exogenous 3,4-AHBA, as well as other 2-aminophenol derivatives^94^. However, *N*-acetylated 3,4-AHBA was not detected under grixazone-producing conditions^95^.

Closer to the more familiar NAT-catalyzed acyl-CoA mediated acyltransfer reaction is the activity of seven NAT homologues in Table 4. Among them, the *ptnC* and *ptmC* genes of *Streptomyces platensis* encode NAT enzymes that can employ (thio)platensicyl- or (thio)platencinyl-CoA as donor substrates, catalyzing the last step in the biosynthesis of antibiotics platencin, platencimycin, and their thiocarboxylic congeners. More specifically, those enzymes form the amide bond which connects the ketolide with the 3-amino-2,4-dihydroxybenzoic acid moiety of the aforementioned products^82–84^. Another example is the *nybK* gene of *Streptomyces albus*, encoding a NAT homologue involved in biosynthesis of the antibiotic nybomycin, where it performs transfer of two acetoacetyl groups from CoA to 2,6-diaminophenol^85^. Acetoacetyl-CoA has been reported to serve as donor substrate for (MYCTU)NAT1 of *Mycobacterium tuberculosis*, but this particular homologue was shown to be part of a cholesterol catabolic gene cluster essential for microbial survival inside macrophages^27^. Furthermore, the *NAT* homologues *daqS* and *daqT* (Table 4) participate in the biosynthesis of diazaquinomycin antibiotics, transferring β-ketoacyl units from CoA to the amine groups of 2,6-diaminohydroquinone^86^. Deviating from the aforementioned acyl-transfer reactions, where the acceptor substrate is an aromatic amine, the product of *cetD* gene (Table 4) performs *N*-acetylation of an aminocyclitol during biosynthesis of the antitumor agent cetoniacytone A^87,88^.

Finally, some BGC-associated NAT homologues have been described to exert *O*-acyltransferase activity towards the hydroxyl group of acceptor substrates (Table 4). For instance, the *tubG* gene of the proteobacterium *Archangium disciforme* is located in the cluster responsible for biosynthesis of the cytotoxin tubulysin, where it encodes a NAT homologue that is proposed to *O*-acylate the pre-tubulysin molecule^90^. Similarly, in *Streptomyces* sp. RI18, the NAT product of *bezG* gene may *O*-acetylate *p*-hydroxyaminobenzoic acid, during the biosynthesis of benzastatins^91^.

## Concluding remarks

Over the past twenty years, we have witnessed progress in genomics by researching the distribution of *NAT* homologues across the entire spectrum of (sequenced) prokaryotic and eukaryotic life^2,3,32,33^ and annotating new *NAT* genes on behalf of the NAT committee^96^. The present study is estimated to have surveyed over 300,000 sequenced microbial genomes and, although this number has almost doubled today, we believe that our portrayal of microbial *NAT* gene distribution, diversity and phylogeny is now comprehensive and unlikely to change substantially. Similarly exciting has been the progression of knowledge about the functional divergence of microbial NATs, captured by many research groups^97^ demonstrating multiple roles of NATs in xenobiotic, secondary and fatty acid metabolic pathways that arm bacteria and fungi to survive or modify their chemical environment and thrive within animal or plant hosts. Given the broad spectrum of functions attributed to microbial NAT enzymes, it is no wonder that scientists have been unable to connect all those homologues under the same consensus nomenclature. Modern databases are nowadays overcoming this difficulty, enabling literature searches using the sequence or other standardized identifiers of genes, proteins and families, while also providing accurate predictions of possible functions. Through the use of such tools, our knowledge of the different roles of NATs in microbes is expanding and the worlds of xenobiotic and secondary metabolism are converging, as recently demonstrated by a group of medicinal chemists characterizing the (STRPT)NAT1 (PtmC) homologue from *Streptomyces platensis* and comparing it with other NATs^84^.

Overall, the experimental evidence supports that the NAT activities associated with bacterial biosynthesis of secondary metabolites can be classified into two main types. The first is the amide synthase activity involved in the production of polyketide ansamycins, while the second is the acyltransferase activity encountered in the biosynthetic pathways of various polyketides, terpenes and other compounds. The association of *NAT* homologues with secondary metabolism is less evident for eukaryotic microorganisms, although *NAT* genes were predicted to participate in clusters relevant to other functions, in line with previous observations. It is also significant that, like other genes of xenobiotic and secondary metabolism, *NAT* sequences are associated with mobile genetic elements involved in HGT, consistent with the mosaic phylogenetic pattern observed for bacterial NATs.

Through our comparative application of different antiSMASH versions, we have been able to follow the advancement of this valuable computational tool. More importantly, the in silico predictions and the experimental findings of the literature retrieved via the MIBiG portal, revealed the extraordinary functional diversification of microbial NAT enzymes in the biosynthesis of secondary metabolites, prompting further research into the role of *NAT* genes in computationally predicted BGCs with as yet uncharacterized functions.

## Methods

### Genomic survey and annotation of microbial *NAT* homologues

*NAT* genes were mined from sequenced microbial genomes and annotated according to established criteria, as previously described^32,33,96^. Searches of the Genome database, accessed through the National Center for Biotechnology Information (NCBI, https://www.ncbi.nlm.nih.gov/genome), were carried out using the tBLASTn algorithm with the appropriate reference sequence as query^33^. Specifically, genomes were interrogated with the following annotated amino acid sequences: (SALTY)NAT1 (GenBank ID: BAA14331.1) of *Salmonella enterica* subsp. *enterica* serovar *Typhimurium* str. LT2 for bacteria; (HALBP)NAT1 (GenBank ID: CBL43355.1) of *Halogeometricum borinquense* str. DSM 11551 for archaea; (GIBMO)NAT1 (GenBank ID: ACD88491.1) for fungi; (DICDI)NAT1 (GenBank ID: CBL43356.1) of *Dictyostelium discoideum* str. AX4 for protists. More focused searches were additionally performed, as necessary, using annotated *NAT* sequences found in microorganisms more closely related to each interrogated taxon. Reconstruction of *NAT* ORFs was performed computationally and/or manually, guided by individual GenBank entries, and annotation was based on inspection of the corresponding translated sequences for identification of the characteristic semi-conserved motifs “VPFENL”, “RGGYC”, “THRL” and “VDV”, where underlined residues indicate the Cys-His-Asp catalytic triad. Species-specific *NAT* gene symbols were assigned based on the percent identity of translated sequences with the corresponding reference sequence mentioned above, according to the guidelines of the *NAT* Gene Nomenclature Committee (http://nat.mbg.duth.gr/)^96,98^. Sequence handling was performed on BioEdit Sequence Alignment Editor 7.0.5.3^99^ and Unipro UGENE^100^.

### Microbial genome mining for BGCs with *NAT* genes

Computational investigation into the possible localization of microbial *NAT* homologues within BGCs was conducted using antiSMASH (https://antismash.secondarymetabolites.org/)^34^. The genomic coordinates of annotated microbial *NAT* genes were initially determined, in order to define the surrounding region. Prokaryotic *NAT* genes were then retrieved together with 500 kb of upstream and downstream flanking sequences (~ 1 Mb of total sequence length), whereas for eukaryotic *NAT* genes the flanking sequences were 1 Mb each (~ 2 Mb in total). Sequences were downloaded in full GenBank format with gene annotations incorporated as provided by the database. Those files were then uploaded to the antiSMASH platform version 3.0^101^, enabling the ClusterFinder algorithm option. The initial analyses were performed in 2016–2018 and were repeated with a larger dataset in 2020, using antiSMASH updated version 5.0^102^ with default parameters. The results were finally validated in 2023, using the new antiSMASH version 7.0^103^. When a *NAT* gene was found within the overlapping region of more than one protocluster, it was considered as part of all protoclusters sharing this region. It is also noted that, newer antiSMASH versions (5.0 and 7.0) fail to run the analysis, if the input sequence begins or terminates with a partial (truncated) ORF. Given the high gene density of microbial genomes, the input sequences thus required additional editing with Unipro UGENE, to remove any partial ORFs from the ends. The GenBank files of all putative clusters containing *NAT* genes were finally downloaded and saved as individual files compiling a comprehensive local dataset. The predictions and BGC definitions with the newer version 7.0 should be regarded as more accurate and complete compared with the previous versions.

### Interrogation of the MIBiG database for BGCs bearing *NAT* genes

For *NAT* genes predicted by antiSMASH to localize within BGCs, the minimum information about a biosynthetic gene cluster (MIBiG, https://mibig.secondarymetabolites.org/)^104^ version 2.0 database was interrogated for previous publications associating NATs with experimentally characterized gene clusters. The content of the MIBiG database was initially downloaded in a FASTA file format. This file, containing all the amino acid sequences encoded by genes from MIBiG entries, was converted into a local database suitable for interrogation via the BLASTp algorithm, using the amino acid sequences of (SALTY)NAT1 or (GIBMO)NAT1 as query. When a *NAT* gene was found within the overlapping region of more than one protocluster, it was considered as part of all the protoclusters sharing this region. The accession numbers of BGC regions identified to harbour *NAT* genes were used to extract additional information regarding the experimental *vs.* computational characterization of the corresponding clusters through the MIBiG repository (https://mibig.secondarymetabolites.org/repository). MIBiG searches were also performed by selecting the MIBiG cluster comparison option in the newer antiSMASH versions (5.0–7.0) employed^56^.

### Search for homology across genomic clusters with *NAT* genes

To assess homology between identified clusters with *NAT* genes, a custom database was first constructed using the cluster sequences in GenBank format. Searches were carried out with the MultiGeneBlast tool^105^, using the GenBank file of each gene cluster of interest as query. Based on the output of each individual search, a multi-sequence FASTA file was created, incorporating all the amino acid sequences encoded by genes found in homologous gene clusters. To visualize those results, this file was then used as query in SimpleSynteny version 1.4 software^106^ and the analysis was performed against a local database comprising the nucleotide sequence FASTA files of the corresponding gene clusters. To avoid redundancies, syntenic units demonstrating 100% conservation were grouped and represented by a single genomic sequence in graphical displays. All procedures were carried out with default program parameters.

### Construction of phylogenetic trees and sequence similarity networks (SSNs)

For the construction of phylogenetic trees, a multiple protein sequence alignment was initially performed on ClustalW^107^. Phylogenetic trees were constructed with MEGAX^108,109^, using neighbor-joining^110^ or maximum likelihood^111^ methods with default parameters. The bootstrap replication number was set to 1000^112^. Common trees for microbial taxa were generated in PHYLIP format using the Common Taxonomy Tree tool of the NCBI (https://www.ncbi.nlm.nih.gov/Taxonomy/CommonTree/wwwcmt.cgi). Generated phylogenetic trees were visualized using the Interactive Tree of Life (iTOL) online resource (https://itol.embl.de/)^113^.

For the construction of SSNs, a FASTA file was created with all protein sequences of interest and an all-by-all BLAST analysis was executed using the EFI-enzyme similarity tool (EFI-EST; https://efi.igb.illinois.edu/efi-est/)^114^, setting the alignment score threshold (E-value) appropriately. The SSN was created by EFI-EST and visualized in Cytoscape^115^. In each SSN, the nodes represent individual proteins and the edges connect nodes when similarity is above the alignment score threshold set for the analysis.

### Search for localization of *NAT* genes in bacterial plasmids

Sequenced bacterial plasmids were accessed via the PLSDB database in 2021 (https://ccb-microbe.cs.uni-saarland.de/plsdb/)^48^, using (SALTY)NAT1 amino acid sequence (GenBank ID: BAA14331.1) as query. Decreasing the High Scoring Pair (HSP) threshold value to as low as 40% retrieved the maximum number of non-redundant tBLASTn hits, which were then analysed and annotated as described above for other *NAT* homologues. Additional information was available through the PLSDB database, e.g., regarding surrounding genes on the same plasmid, the microbiological sample of origin, etc. The identified plasmid sequences were subsequently subjected to antiSMASH (version 6.0) search for BGCs, activating the MIBiG cluster comparison option. The specific features of plasmid BGCs with *NAT* genes were then recorded. The plasmids were further screened using IslandViewer version 4 (https://www.pathogenomics.sfu.ca/islandviewer/)^116,117^ for putative genomic islands, and those were inspected for the presence of *NAT* genes within them.

### Supplementary Information

Supplementary Information 1.

Supplementary Information 2.

Supplementary Information 3.

Supplementary Information 4.

Supplementary Information 5.

Supplementary Information 6.

Supplementary Information 7.

Supplementary Information 8.

Supplementary Information 9.

## Data Availability

All data generated or analysed during this study are included in this published article (and its Supplementary Information files).

## Abbreviations

3,4-AHBA: 3-Amino-4-hydroxybenzoic acid
3,5-AHBA: 3-Amino-5-hydroxybenzoic acid
antiSMASH: Secondary metabolite analysis shell software
BGC: Biosynthetic gene cluster
CoA: Coenzyme A
EFI-EST: EFI-enzyme similarity tool
HGT: Horizontal gene transfer
MIBiG: Minimum information about a biosynthetic gene cluster
NRPS: Non-ribosomal peptide synthase
ORF: Open reading frame
PKS: Polyketide synthase
SSN: Sequence similarity network
