# Supplementary material for: Insights into the genomic and functional divergence of NAT gene family to serve microbial secondary metabolism
Source: Sci Rep. 2024 Jun 28;14:14905. doi: 10.1038/s41598-024-65342-4 (PMC11213898; doi:10.1038/s41598-024-65342-4)
Supplement: Supplementary file 4 — Supplementary Information 4. [file 41598_2024_65342_MOESM4_ESM.pdf]

Boukouvala et al.:

Insights into the genomic and functional divergence of *NAT* gene family to serve microbial secondary metabolism

Supplementary Information S4:

Illustration of the synteny between putative clusters with *NAT* genes in mycobacteria, predicted by antiSMASH 3.0 and MultiGeneBlast, and visualized in SimpleSynteny 1.4.

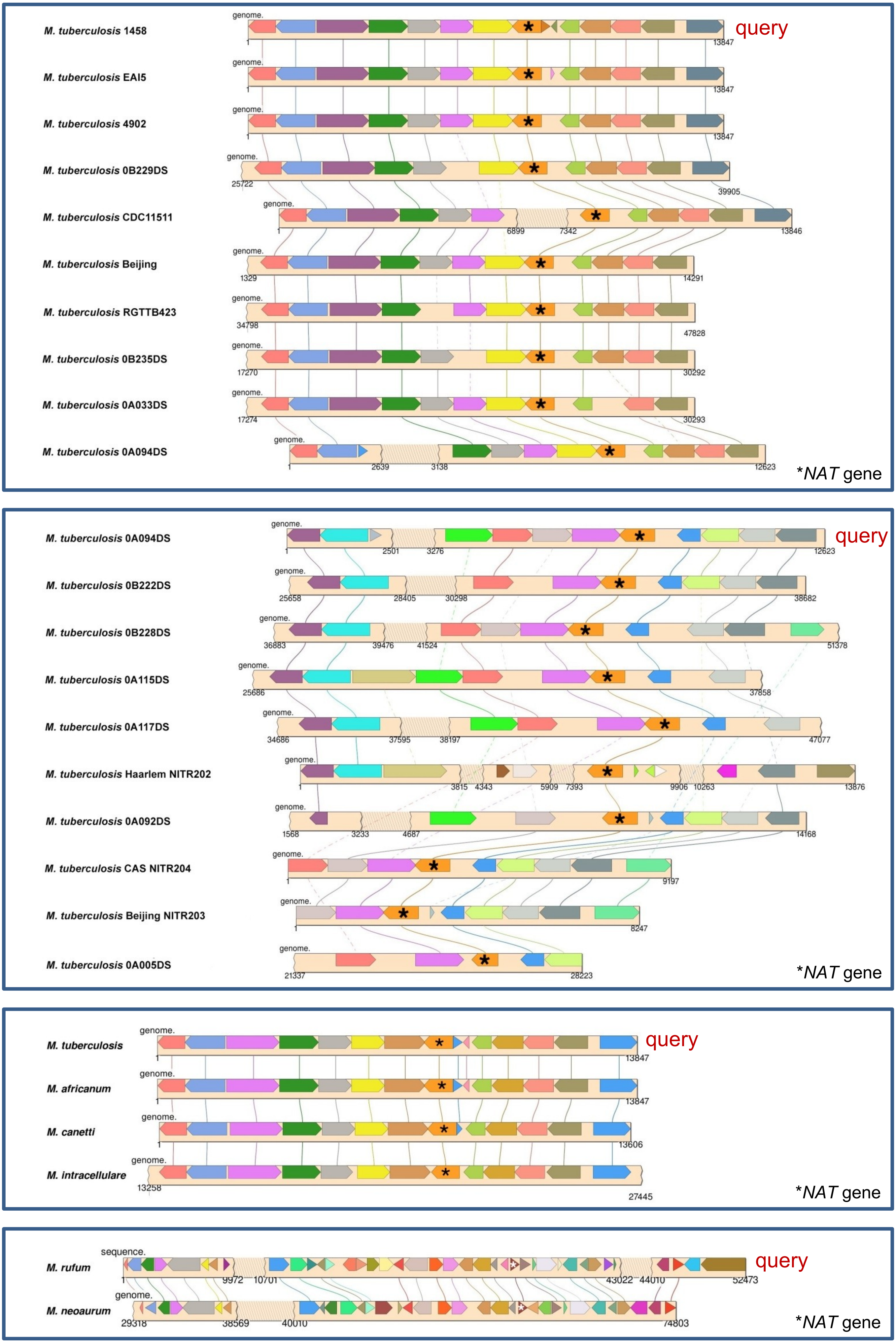

M. tuberculosis 1458
M. tuberculosis EAI5
M. tuberculosis 4902
M. tuberculosis 0B229DS
M. tuberculosis CDC11511
M. tuberculosis Beijing
M. tuberculosis RGTTB423
M. tuberculosis 0B235DS
M. tuberculosis 0A033DS
M. tuberculosis 0A094DS
query
*NAT gene
genome.
1
13847
25722
39905
6899
7342
13846
1329
14291
34798
47828
17270
30292
17274
30293
2639
3138
12623
M. tuberculosis 0A094DS
M. tuberculosis 0B222DS
M. tuberculosis 0B228DS
M. tuberculosis 0A115DS
M. tuberculosis 0A117DS
M. tuberculosis Haarlem NITR202
M. tuberculosis 0A092DS
M. tuberculosis CAS NITR204
M. tuberculosis Beijing NITR203
M. tuberculosis 0A005DS
query
*NAT gene
2501
3276
12623
25658
28405
30298
38682
36883
39476
41524
51378
25686
37858
34686
37595
38197
47077
3815
4343
5909
7393
9906
10263
13876
1568
3233
4687
14168
9197
8247
21337
28223
M. tuberculosis
M. africanum
M. canetti
M. intracellulare
query
*NAT gene
13847
13606
13258
27445
M. rufum
M. neoaurum
sequence.
query
*NAT gene
9972
10701
43022
44010
52473
29318
38569
40010
74803
