# Supplementary material for: Insights into the genomic and functional divergence of NAT gene family to serve microbial secondary metabolism
Source: Sci Rep. 2024 Jun 28;14:14905. doi: 10.1038/s41598-024-65342-4 (PMC11213898; doi:10.1038/s41598-024-65342-4)
Supplement: Supplementary file 5 — Supplementary Information 5. [file 41598_2024_65342_MOESM5_ESM.pdf]

Boukouvala et al.:
Insights into the genomic and functional divergence of *NAT* gene family to serve microbial secondary metabolism

Supplementary Information S5:

Representative illustrations of synteny between putative clusters with *NAT* genes in actinobacteria. S5a is an example of NRPS cluster synteny between different strains of the same species (*Streptomyces rimosus*), while S5b shows synteny of a terpene cluster between different species of the same genus (*Pseudonocardia*).

S5a

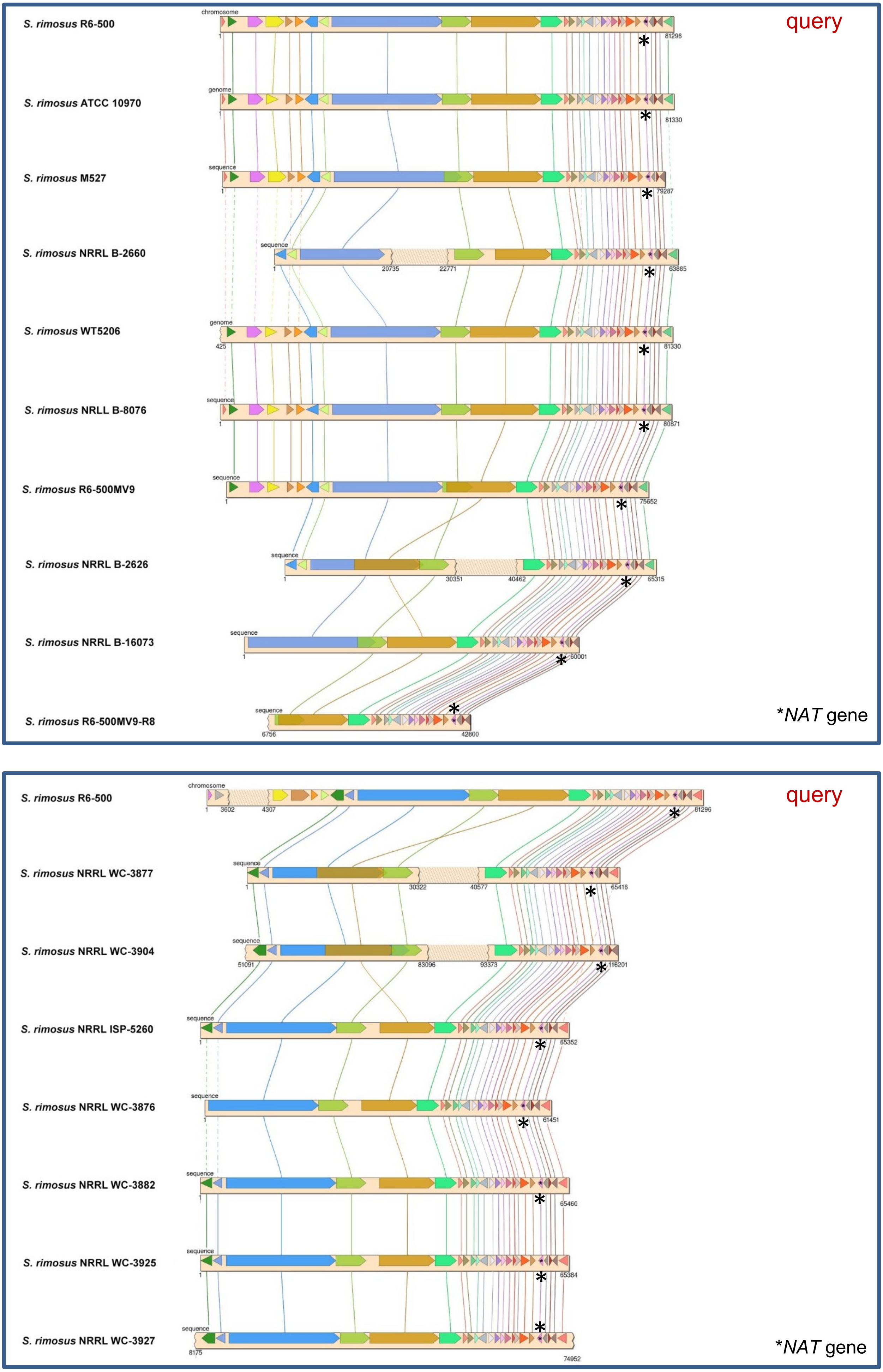

S. rimosus R6-500
chromosome
81296
query
S. rimosus ATCC 10970
genome
81330
S. rimosus M527
sequence
79287
S. rimosus NRRL B-2660
sequence
20735
22771
63885
S. rimosus WT5206
genome
425
81330
S. rimosus NRLL B-8076
sequence
80871
S. rimosus R6-500MV9
sequence
75652
S. rimosus NRRL B-2626
sequence
30351
40462
65315
S. rimosus NRRL B-16073
sequence
60001
S. rimosus R6-500MV9-R8
sequence
6756
42800
*NAT gene
S. rimosus R6-500
chromosome
3602
4307
81296
query
S. rimosus NRRL WC-3877
sequence
30322
40577
65416
S. rimosus NRRL WC-3904
sequence
51091
83096
93373
116201
S. rimosus NRRL ISP-5260
sequence
65352
S. rimosus NRRL WC-3876
sequence
61451
S. rimosus NRRL WC-3882
sequence
65460
S. rimosus NRRL WC-3925
sequence
65384
S. rimosus NRRL WC-3927
sequence
8175
74952
*NAT gene

S5b

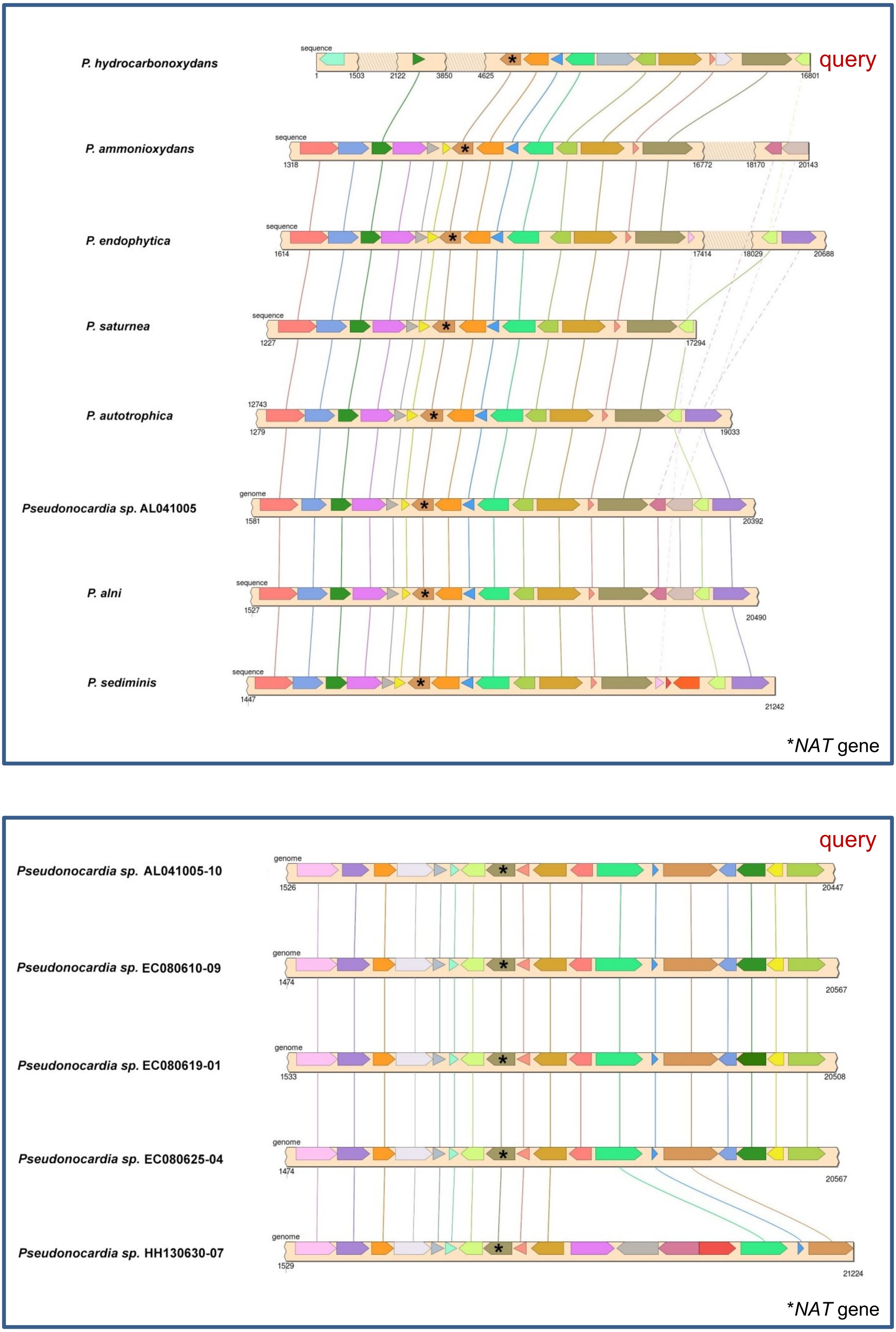
P. hydrocarbonoxydans
sequence
1
1503
2122
3850
4625
16801
query
P. ammonioxydans
sequence
1318
16772
18170
20143
P. endophytica
sequence
1614
17414
18029
20688
P. saturnea
sequence
1227
17294
P. autotrophica
12743
1279
19033
Pseudonocardia sp. AL041005
genome
1581
20392
P. alni
sequence
1527
20490
P. sediminis
sequence
1447
21242
*NAT gene
query
Pseudonocardia sp. AL041005-10
genome
1526
20447
Pseudonocardia sp. EC080610-09
genome
1474
20567
Pseudonocardia sp. EC080619-01
genome
1533
20508
Pseudonocardia sp. EC080625-04
genome
1474
20567
Pseudonocardia sp. HH130630-07
genome
1529
21224
*NAT gene
