# Supplementary material for: Insights into the genomic and functional divergence of NAT gene family to serve microbial secondary metabolism
Source: Sci Rep. 2024 Jun 28;14:14905. doi: 10.1038/s41598-024-65342-4 (PMC11213898; doi:10.1038/s41598-024-65342-4)
Supplement: Supplementary file 6 — Supplementary Information 6. [file 41598_2024_65342_MOESM6_ESM.pdf]

Boukouvala et al.:
Insights into the genomic and functional divergence of *NAT* gene family to serve microbial secondary metabolism

Supplementary Information S6:

Distribution of *NAT* genes per prokaryotic taxon and type of biosynthetic gene cluster (BGC), determined during the antiSMASH 5.0 analyses (including MIBiG). The species tree is presented in S6a, constructed according to conventional taxonomy (NCBI Taxonomy Database common tree), and the leaves are coloured as shown in the figure key. The phylogenetic tree of BGC-associated NAT sequences is presented in S6b, constructed using the neighbour-joining method, and the leaves are coloured according to taxonomy. The phylogenetic tree of S6b is also presented in S6c, with leaves coloured according to cluster type. The corresponding NAT trees generated with the maximum likelihood method are also provided in S6d and S6e. Note that the streptomycetes are shown with a different colour from other actinobacteria.

[Note: S6a-c are the same as in the main manuscript, shown here enlarged for additional clarity]

S6a

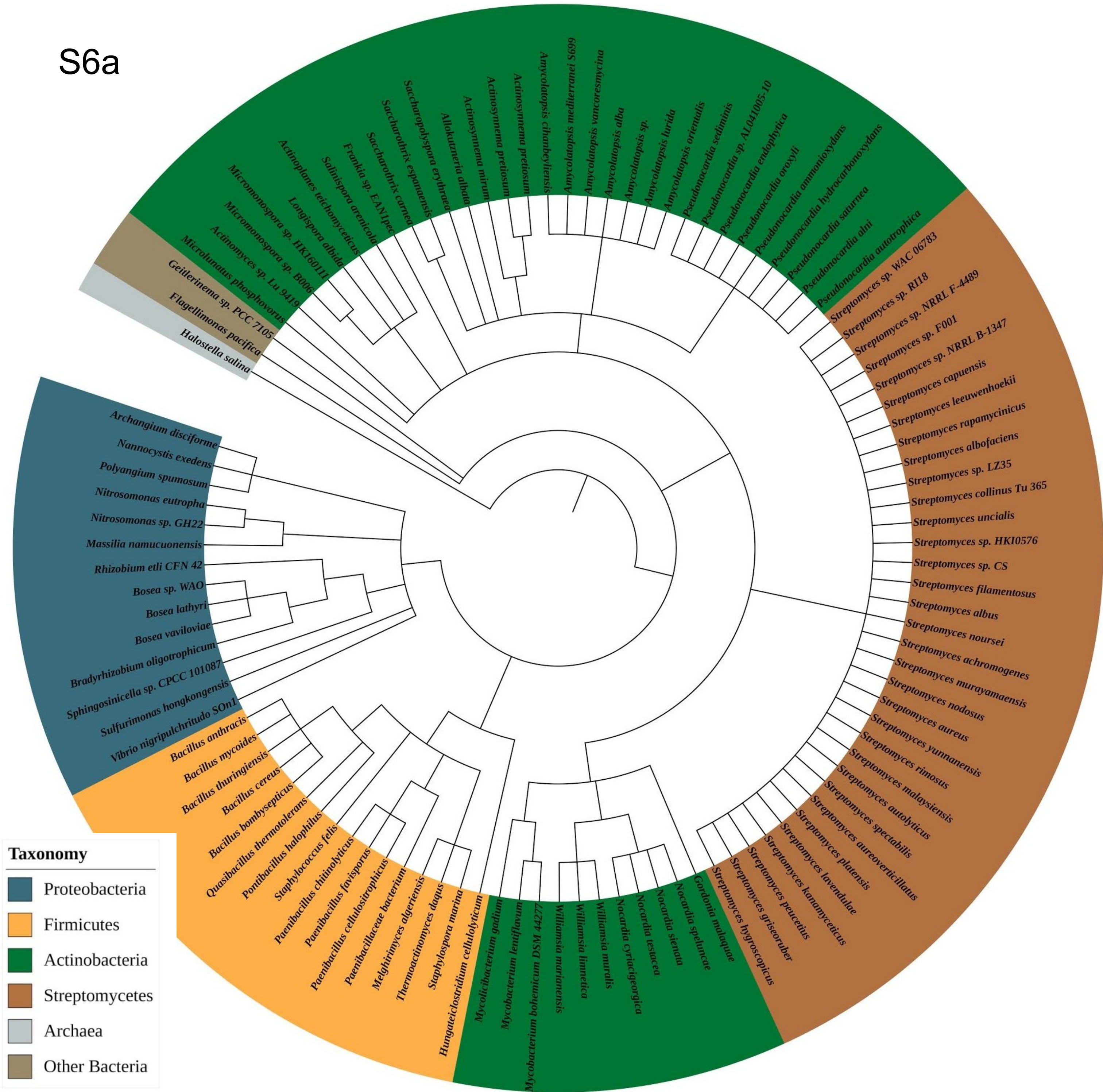
Archangium disciforme
Nannocystis exedens
Polyangium spumosum
Nitrosomonas eutropha
Nitrosomonas sp. GH22
Massilia namucuonensis
Rhizobium etli CFN 42
Bosea sp. WAO
Bosea lathyri
Bosea vaviloviae
Bradyrhizobium oligotrophicum
Sphingosinicella sp. CPCC 101087
Sulfurimonas hongkongensis
Vibrio nigripulchritudo SOn1
Bacillus anthracis
Bacillus mycoides
Bacillus thuringiensis
Bacillus cereus
Bacillus bombysepticus
Quasibacillus thermotolerans
Pontibacillus halophilus
Staphylococcus felis
Paenibacillus chitinolyticus
Paenibacillus favisporus
Paenibacillus cellulositrophicus
Paenibacillaceae bacterium
Melghirimyces algeriensis
Thermoactinomyces daqus
Staphylospora marina
Hungateiclostridium cellulolyticum
Mycolicibacterium gadium
Mycobacterium lentiflavum
Mycobacterium bohemicum DSM 44277
Williamsia marianensis
Williamsia limnetica
Williamsia muralis
Nocardia cyriacigeorgica
Nocardia testacea
Nocardia sienata
Nocardia speluncae
Gordonia malaquae
Streptomyces hygroscopicus
Streptomyces griseoruber
Streptomyces peucetius
Streptomyces kanamyceticus
Streptomyces lavendulae
Streptomyces platensis
Streptomyces aureoverticillatus
Streptomyces spectabilis
Streptomyces autolyticus
Streptomyces malaysiensis
Streptomyces rimosus
Streptomyces yunnanensis
Streptomyces aureus
Streptomyces nodosus
Streptomyces murayamaensis
Streptomyces achromogenes
Streptomyces noursei
Streptomyces albus
Streptomyces filamentosus
Streptomyces sp. CS
Streptomyces sp. HKI0576
Streptomyces uncialis
Streptomyces collinus Tu 365
Streptomyces sp. LZ35
Streptomyces albofaciens
Streptomyces rapamycinicus
Streptomyces leeuwenhoekii
Streptomyces capuensis
Streptomyces sp. NRRL B-1347
Streptomyces sp. F001
Streptomyces sp. NRRL F-4489
Streptomyces sp. RI18
Streptomyces sp. WAC 06783
Pseudonocardia autotrophica
Pseudonocardia alni
Pseudonocardia saturnea
Pseudonocardia hydrocarbonoxydans
Pseudonocardia ammonioxydans
Pseudonocardia oroxyli
Pseudonocardia endophytica
Pseudonocardia sp. AL041005-10
Pseudonocardia sediminis
Amycolatopsis orientalis
Amycolatopsis lurida
Amycolatopsis sp.
Amycolatopsis alba
Amycolatopsis vancoresmycina
Amycolatopsis mediterranei S699
Amycolatopsis cihanbeyliensis
Actinosynnema pretiosum
Actinosynnema pretiosum
Actinosynnema mirum
Allokutzneria albata
Saccharopolyspora erythraea
Saccharothrix espanaensis
Saccharothrix carnea
Frankia sp. EAN1pec
Salinispora arenicola
Actinoplanes teichomyceticus
Longispora albida
Micromonospora sp. HK160111
Micromonospora sp. B006
Actinomyces sp. Lu 9419
Microlunatus phosphovorus
Geitlerinema sp. PCC 7105
Flagellimonas pacifica
Halostella salina
Taxonomy
Proteobacteria
Firmicutes
Actinobacteria
Streptomycetes
Archaea
Other Bacteria

S6b

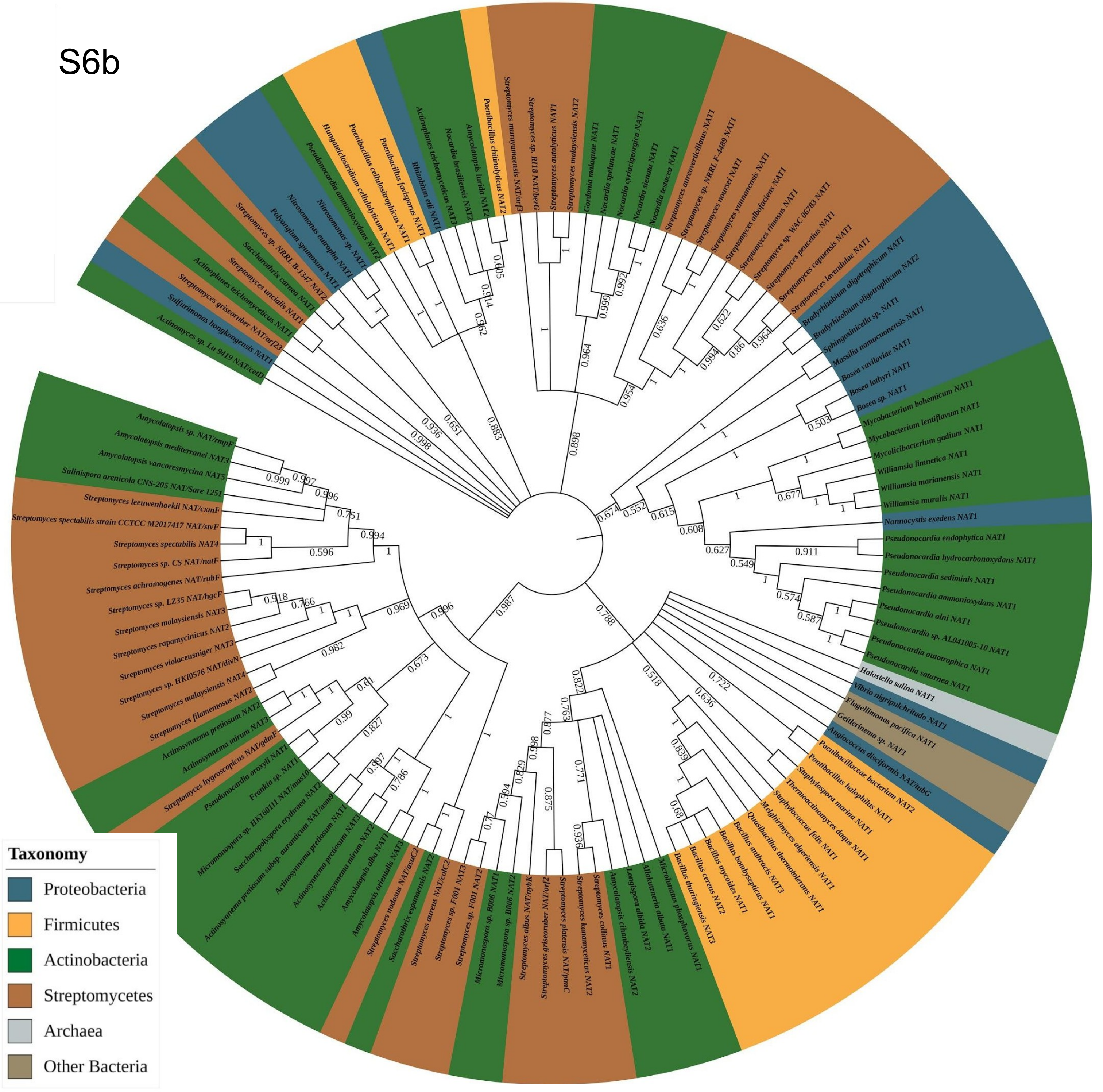
Taxonomy
Proteobacteria
Firmicutes
Actinobacteria
Streptomycetes
Archaea
Other Bacteria
Actinomyces sp. Lu 9419 NAT/cetD
Sulfurimonas hongkongensis NAT1
Streptomyces griseoruber NAT/orf23
Actinoplanes teichomyceticus NAT1
Streptomyces uncialis NAT1
Saccharothrix carnea NAT1
Streptomyces sp. NRRL B-1347 NAT2
Polyangium spumosum NAT1
Nitrosomonas europha NAT1
Nitrosomonas sp. NAT1
Pseudonocardia ammonioxydans NAT2
Hungateiclostridium cellulolyticum NAT1
Paenibacillus cellulositrophicus NAT1
Paenibacillus favisporus NAT1
Rhizobium etli NAT1
Actinoplanes teichomyceticus NAT3
Nocardia brasiliensis NAT2
Amycolatopsis lurida NAT2
Paenibacillus chitinolyticus NAT2
Streptomyces murayamaensis NAT/orf3
Streptomyces sp. RI18 NAT/hezG
Streptomyces autolyticus NAT1
Streptomyces malaysiensis NAT2
Gordonia malaquae NAT1
Nocardia speluncae NAT1
Nocardia cyriacigeorgica NAT1
Nocardia sienata NAT1
Nocardia testacea NAT1
Streptomyces aureoverticillatus NAT1
Streptomyces sp. NRRL F-4489 NAT1
Streptomyces noursei NAT1
Streptomyces yunnanensis NAT1
Streptomyces albofaciens NAT1
Streptomyces rimosus NAT1
Streptomyces sp. WAC 06783 NAT1
Streptomyces peucetius NAT1
Streptomyces capuensis NAT1
Streptomyces lavendulae NAT1
Bradyrhizobium oligotrophicum NAT1
Bradyrhizobium oligotrophicum NAT2
Sphingosinicella sp. NAT1
Massilia namucuonensis NAT1
Bosea vaviloviae NAT1
Bosea lathyri NAT1
Bosea sp. NAT1
Mycobacterium bohemicum NAT1
Mycobacterium lentiflavum NAT1
Mycolicibacterium gadium NAT1
Williamsia limnetica NAT1
Williamsia marianensis NAT1
Williamsia muralis NAT1
Nannocystis exedens NAT1
Pseudonocardia endophytica NAT1
Pseudonocardia hydrocarbonoxydans NAT1
Pseudonocardia sediminis NAT1
Pseudonocardia ammonioxydans NAT1
Pseudonocardia alni NAT1
Pseudonocardia sp. AL041005-10 NAT1
Pseudonocardia autotrophica NAT1
Pseudonocardia saturnea NAT1
Halostella salina NAT1
Vibrio nigripulchritudo NAT1
Flagellimonas pacifica NAT1
Geitlerinema sp. NAT1
Angiococcus disciformis NAT/tubG
Paenibacillaceae bacterium NAT2
Pontibacillus halophilus NAT1
Staphylospora marina NAT1
Thermoactinomyces daqus NAT1
Staphylococcus felis NAT1
Melghirimyces algeriensis NAT1
Quasibacillus thermotolerans NAT1
Bacillus anthracis NAT3
Bacillus bombysepticus NAT1
Bacillus mycoides NAT1
Bacillus cereus NAT2
Bacillus thuringiensis NAT3
Microlunatus phosphovorus NAT1
Allokutzneria albata NAT1
Longispora albida NAT2
Amycolatopsis cihanbeyliensis NAT2
Streptomyces collinus NAT1
Streptomyces kanamyceticus NAT2
Streptomyces platensis NAT/pmC
Streptomyces griseoruber NAT/orf2
Streptomyces albus NAT/nybK
Micromonospora sp. B006 NAT2
Micromonospora sp. B006 NAT1
Streptomyces sp. F001 NAT2
Streptomyces sp. F001 NAT3
Streptomyces aureus NAT/colC2
Saccharothrix espanaensis NAT2
Streptomyces nodosus NAT/asuC2
Amycolatopsis orientalis NAT3
Amycolatopsis alba NAT1
Actinosynnema mirum NAT2
Actinosynnema pretiosum NAT3
Actinosynnema pretiosum NAT1
Actinosynnema pretiosum subsp. auranticum NAT/asm9
Saccharopolyspora erythraea NAT2
Micromonospora sp. HK160111 NAT/mas10
Frankia sp. NAT1
Pseudonocardia oroxyli NAT1
Streptomyces hygroscopicus NAT/gdmF
Actinosynnema mirum NAT3
Actinosynnema pretiosum NAT2
Streptomyces filamentosus NAT2
Streptomyces malaysiensis NAT4
Streptomyces sp. HKI0576 NAT/divN
Streptomyces violaceusniger NAT3
Streptomyces rapamycinicus NAT2
Streptomyces malaysiensis NAT3
Streptomyces sp. LZ35 NAT/hgcF
Streptomyces achromogenes NAT/rubF
Streptomyces sp. CS NAT/natF
Streptomyces spectabilis NAT4
Streptomyces spectabilis strain CCTCC M2017417 NAT/stvF
Streptomyces leeuwenhoekii NAT/cxmF
Salinispora arenicola CNS-205 NAT/Sare 1251
Amycolatopsis vancoresmycina NAT3
Amycolatopsis mediterranei NAT3
Amycolatopsis sp. NAT/rmpF

S6c

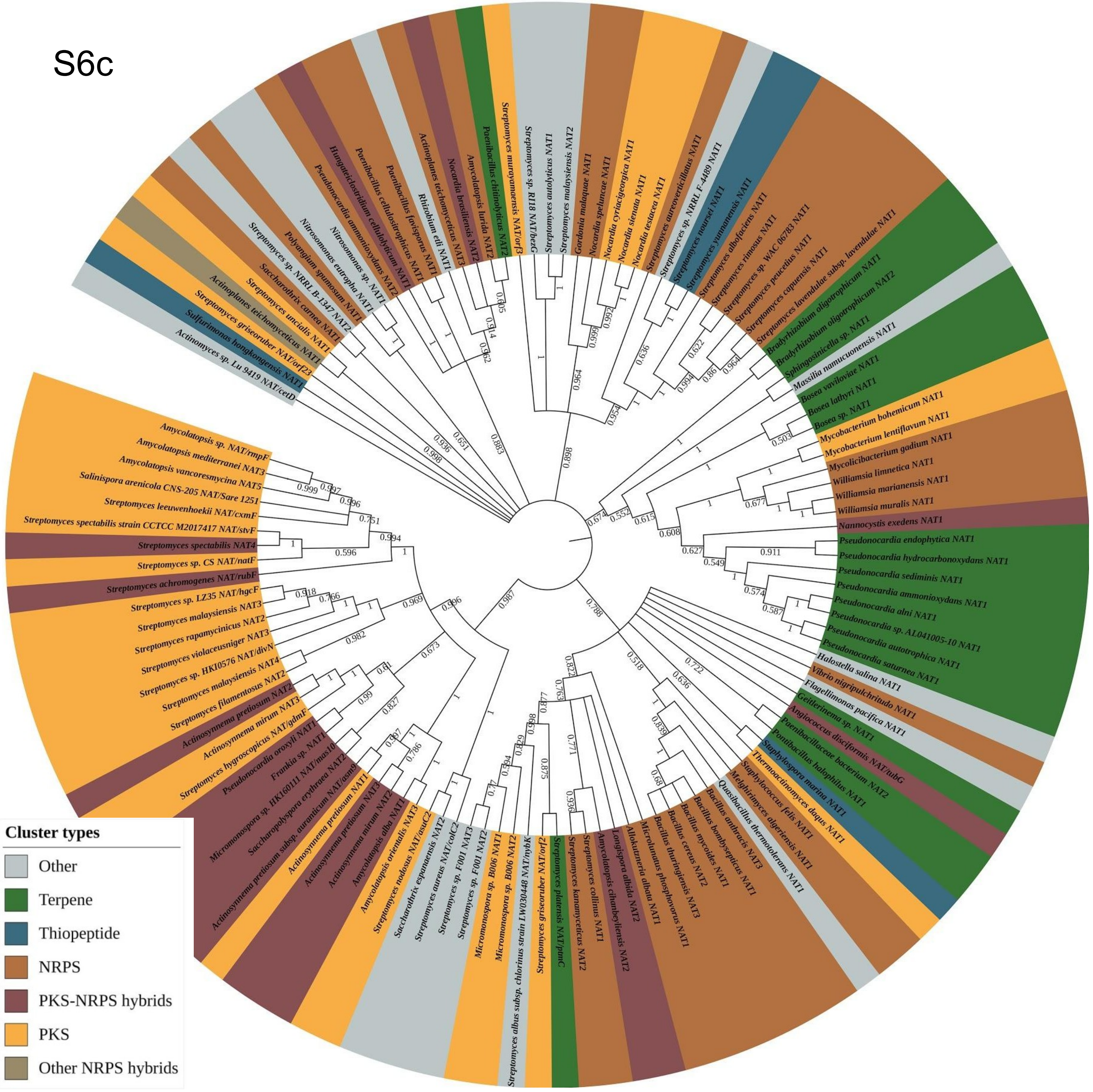

Cluster types
Other
Terpene
Thiopeptide
NRPS
PKS-NRPS hybrids
PKS
Other NRPS hybrids
Actinomyces sp. Lu 9419 NAT/cetD
Sulfurimonas hongkongensis NAT1
Streptomyces griseoruber NAT/orf23
Actinoplanes teichomyceticus NAT1
Streptomyces uncialis NAT1
Saccharothrix carnea NAT2
Streptomyces sp. NRRL B-1347 NAT1
Polyangium spumosum NAT1
Nitrosomonas eutropha NAT1
Nitrosomonas sp. NAT1
Pseudonocardia ammonioxydans NAT2
Hungateiclostridium cellulolyticum NAT1
Paenibacillus cellulositrophicus NAT1
Paenibacillus favisporus NAT1
Rhizobium etli NAT1
Actinoplanes teichomyceticus NAT3
Nocardia brasiliensis NAT2
Amycolatopsis lurida NAT2
Paenibacillus chitinolyticus NAT2
Streptomyces murayamaensis NAT/orf3
Streptomyces sp. RI18 NAT/bezC
Streptomyces autolyticus NAT1
Streptomyces malaysiensis NAT2
Gordonia malaquae NAT1
Nocardia speluncae NAT1
Nocardia cyriacigeorgica NAT1
Nocardia sienata NAT1
Nocardia testacea NAT1
Streptomyces aureoverticillatus NAT1
Streptomyces sp. NRRL F-4489 NAT1
Streptomyces noursei NAT1
Streptomyces yunnanensis NAT1
Streptomyces albofaciens NAT1
Streptomyces rimosus NAT1
Streptomyces sp. WAC 06783 NAT1
Streptomyces peucetius NAT1
Streptomyces capuensis NAT1
Streptomyces lavendulae subsp. lavendulae NAT1
Bradyrhizobium oligotrophicum NAT1
Bradyrhizobium oligotrophicum NAT2
Sphingosinicella sp. NAT1
Massilia namucuonensis NAT1
Bosea vaviloviae NAT1
Bosea lathyri NAT1
Bosea sp. NAT1
Mycobacterium bohemicum NAT1
Mycobacterium lentiflavum NAT1
Mycolicibacterium gadium NAT1
Williamsia limnetica NAT1
Williamsia marianensis NAT1
Williamsia muralis NAT1
Nannocystis exedens NAT1
Pseudonocardia endophytica NAT1
Pseudonocardia hydrocarbonoxydans NAT1
Pseudonocardia sediminis NAT1
Pseudonocardia ammonioxydans NAT1
Pseudonocardia alni NAT1
Pseudonocardia sp. AL041005-10 NAT1
Pseudonocardia autotrophica NAT1
Pseudonocardia saturnea NAT1
Halostella salina NAT1
Vibrio nigripulchritudo NAT1
Flagellimonas pacifica NAT1
Geitlerinema sp. NAT1
Angiococcus disciformis NAT/tubG
Paenibacillaceae bacterium NAT2
Pontibacillus halophilus NAT1
Staphylospora marina NAT1
Thermoactinomyces daqus NAT1
Staphylococcus felis NAT1
Melghirimyces algeriensis NAT1
Quasibacillus thermotolerans NAT1
Bacillus anthracis NAT3
Bacillus bombysepticus NAT1
Bacillus mycoides NAT1
Bacillus cereus NAT2
Bacillus thuringiensis NAT3
Microlunatus phosphovorus NAT1
Allokutzneria albata NAT1
Longispora albida NAT2
Amycolatopsis cihanbeyliensis NAT2
Streptomyces collinus NAT1
Streptomyces kanamyceticus NAT2
Streptomyces platensis NAT/ptmC
Streptomyces griseoruber NAT/orf2
Streptomyces albus subsp. chlorinus strain LW030448 NAT/nybK
Micromonospora sp. B006 NAT2
Micromonospora sp. B006 NAT1
Streptomyces sp. F001 NAT2
Streptomyces sp. F001 NAT3
Streptomyces aureus NAT/colC2
Saccharothrix espanaensis NAT2
Streptomyces nodosus NAT/asuC2
Amycolatopsis orientalis NAT3
Amycolatopsis alba NAT1
Actinosynnema mirum NAT2
Actinosynnema pretiosum NAT3
Actinosynnema pretiosum NAT1
Actinosynnema pretiosum subsp. auranticum NAT/asm8
Saccharopolyspora erythraea NAT2
Micromonospora sp. HK160111 NAT/mas10
Frankia sp. NAT1
Pseudonocardia oroxyli NAT1
Streptomyces hygroscopicus NAT/gdmF
Actinosynnema mirum NAT3
Actinosynnema pretiosum NAT2
Streptomyces filamentosus NAT2
Streptomyces malaysiensis NAT4
Streptomyces sp. HK10576 NAT/divN
Streptomyces violaceusniger NAT3
Streptomyces rapamycinicus NAT2
Streptomyces malaysiensis NAT3
Streptomyces sp. LZ35 NAT/hgcF
Streptomyces achromogenes NAT/rubF
Streptomyces sp. CS NAT/natF
Streptomyces spectabilis NAT4
Streptomyces spectabilis strain CCTCC M2017417 NAT/stvF
Streptomyces leeuwenhoekii NAT/cxmF
Salinispora arenicola CNS-205 NAT/Sare 1251
Amycolatopsis vancoresmycina NAT5
Amycolatopsis mediterranei NAT3
Amycolatopsis sp. NAT/rmpF

S6d

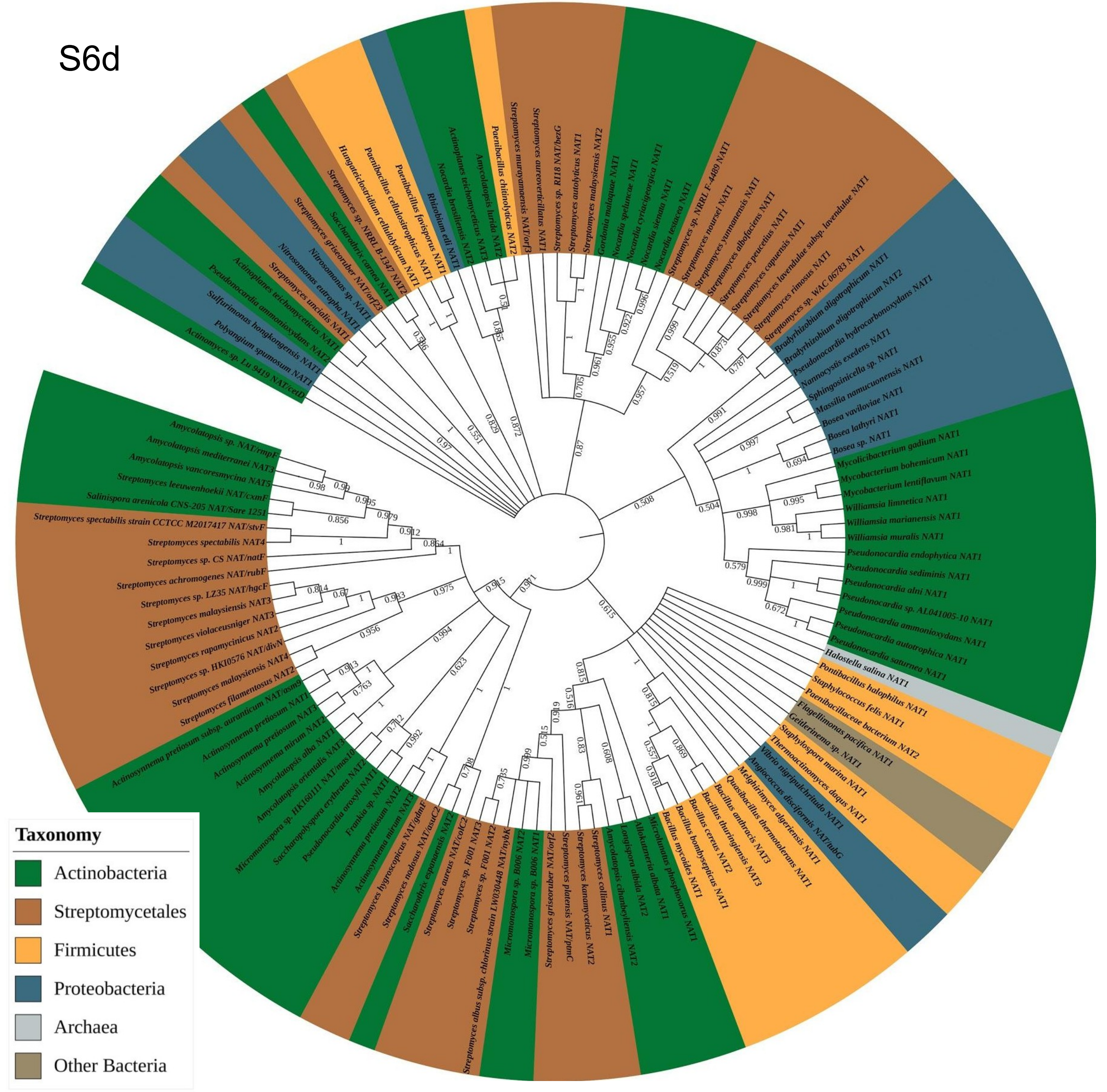
Actinomyces sp. Lu 9419 NAT/cetD
Polyangium spumosum NAT1
Sulfurimonas hongkongensis NAT1
Pseudonocardia ammonioxydans NAT2
Actinoplanes teichomyceticus NAT1
Streptomyces uncialis NAT1
Nitrosomonas europha NAT1
Nitrosomonas sp. NAT1
Streptomyces griseoruber NAT/orf23
Saccharothrix carnea NAT1
Streptomyces sp. NRRL B-1347 NAT2
Hungateiclostridium cellulolyticum NAT1
Paenibacillus cellulositrophicus NAT1
Paenibacillus favisporus NAT1
Rhizobium etli NAT1
Nocardia brasiliensis NAT2
Actinoplanes teichomyceticus NAT3
Amycolatopsis lurida NAT2
Paenibacillus chitinolyticus NAT2
Streptomyces murayamaensis NAT/orf3
Streptomyces aureoverticillatus NAT1
Streptomyces sp. RI18 NAT/bezG
Streptomyces autolyticus NAT1
Streptomyces malaysiensis NAT2
Gordonia malaquae NAT1
Nocardia speluncae NAT1
Nocardia cyriacigeorgica NAT1
Nocardia sienata NAT1
Nocardia testacea NAT1
Streptomyces sp. NRRL F-4489 NAT1
Streptomyces noursei NAT1
Streptomyces yunnanensis NAT1
Streptomyces albofaciens NAT1
Streptomyces peucetius NAT1
Streptomyces capuensis NAT1
Streptomyces lavendulae subsp. lavendulae NAT1
Streptomyces rimosus NAT1
Streptomyces sp. WAC 06783 NAT1
Bradyrhizobium oligotrophicum NAT1
Bradyrhizobium oligotrophicum NAT2
Pseudonocardia hydrocarbonoxydans NAT1
Nannocystis exedens NAT1
Sphingosinicella sp. NAT1
Massilia namucuonensis NAT1
Bosea vaviloviae NAT1
Bosea lathyri NAT1
Bosea sp. NAT1
Mycolicibacterium gadium NAT1
Mycobacterium bohemicum NAT1
Mycobacterium lentiflavum NAT1
Williamsia limnetica NAT1
Williamsia marianensis NAT1
Williamsia muralis NAT1
Pseudonocardia endophytica NAT1
Pseudonocardia sediminis NAT1
Pseudonocardia alni NAT1
Pseudonocardia sp. AL041005-10 NAT1
Pseudonocardia ammonioxydans NAT1
Pseudonocardia autotrophica NAT1
Pseudonocardia saturnea NAT1
Halostella salina NAT1
Pontibacillus halophilus NAT1
Staphylococcus felis NAT1
Paenibacillaceae bacterium NAT2
Flagellimonas pacifica NAT1
Geitlerinema sp. NAT1
Staphylospora marina NAT1
Thermoactinomyces daqus NAT1
Vibrio nigripulchritudo NAT1
Angiococcus disciformis NAT/tubG
Melghirimyces algeriensis NAT1
Quasibacillus thermotolerans NAT1
Bacillus anthracis NAT3
Bacillus thuringiensis NAT3
Bacillus cereus NAT2
Bacillus bombysepticus NAT1
Bacillus mycoides NAT1
Microlunatus phosphovorus NAT1
Allokutzneria albata NAT1
Longispora albida NAT2
Amycolatopsis cihanbeyliensis NAT2
Streptomyces collinus NAT1
Streptomyces kanamyceticus NAT2
Streptomyces platensis NAT/pmC
Streptomyces griseoruber NAT/orf2
Micromonospora sp. B006 NAT1
Micromonospora sp. B006 NAT2
Streptomyces albus subsp. chlorinus strain LW030448 NAT/nybK
Streptomyces sp. F001 NAT2
Streptomyces sp. F001 NAT3
Streptomyces aureus NAT/colC2
Saccharothrix espanaensis NAT2
Streptomyces nodosus NAT/asuC2
Streptomyces hygroscopicus NAT/gdmF
Actinosynnema mirum NAT3
Actinosynnema pretiosum NAT2
Frankia sp. NAT1
Pseudonocardia oroxyli NAT1
Saccharopolyspora erythraea NAT2
Micromonospora sp. HK160111 NAT/mas10
Amycolatopsis orientalis NAT3
Amycolatopsis alba NAT1
Actinosynnema mirum NAT2
Actinosynnema pretiosum NAT3
Actinosynnema pretiosum NAT1
Actinosynnema pretiosum subsp. auranticum NAT/asm9
Streptomyces filamentosus NAT2
Streptomyces malaysiensis NAT4
Streptomyces sp. HKI0576 NAT/divN
Streptomyces rapamycinicus NAT2
Streptomyces violaceusniger NAT3
Streptomyces malaysiensis NAT3
Streptomyces sp. LZ35 NAT/hgcF
Streptomyces achromogenes NAT/rubF
Streptomyces sp. CS NAT/natF
Streptomyces spectabilis NAT4
Streptomyces spectabilis strain CCTCC M2017417 NAT/stvF
Salinispora arenicola CNS-205 NAT/Sare 1251
Streptomyces leeuwenhoekii NAT/cxmF
Amycolatopsis vancoresmycina NAT5
Amycolatopsis mediterranei NAT3
Amycolatopsis sp. NAT/rmpF
Taxonomy
Actinobacteria
Streptomycetales
Firmicutes
Proteobacteria
Archaea
Other Bacteria

S6e

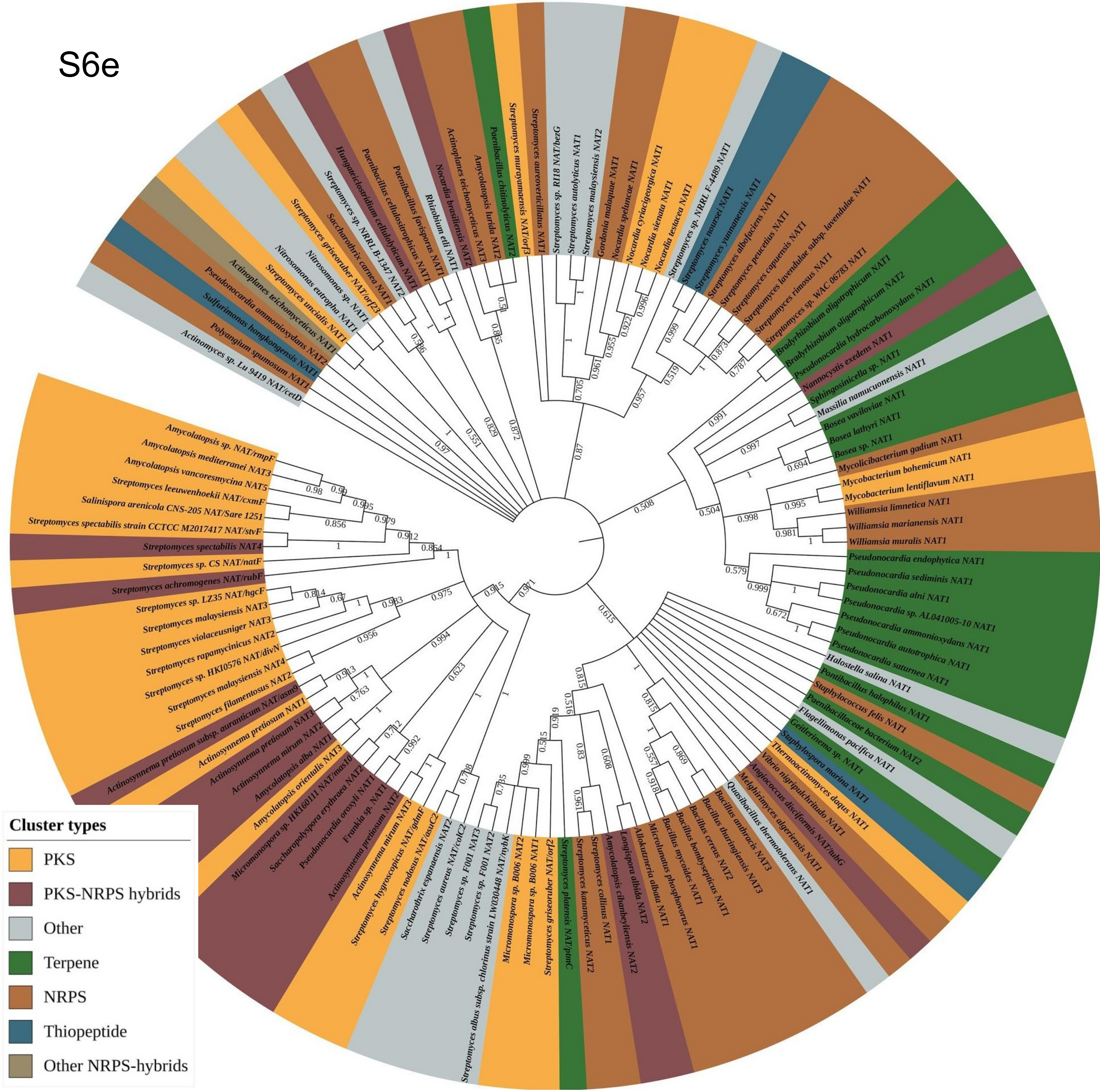
Actinomyces sp. Lu 9419 NAT/cetD
Polyangium spumosum NAT1
Sulfurimonas hongkongensis NAT1
Pseudonocardia ammonioxydans NAT2
Actinoplanes teichomyceticus NAT1
Streptomyces uncialis NAT1
Nitrosomonas eutropha NAT1
Nitrosomonas sp. NAT1
Streptomyces griseoruber NAT/orf23
Saccharothrix carnea NAT1
Streptomyces sp. NRRL B-1347 NAT2
Hungateiclostridium cellulolyticum NAT1
Paenibacillus cellulositrophicus NAT1
Paenibacillus favisporus NAT1
Rhizobium etli NAT1
Nocardia brasiliensis NAT2
Actinoplanes teichomyceticus NAT3
Amycolatopsis lurida NAT2
Paenibacillus chitinolyticus NAT2
Streptomyces murayamaensis NAT/orf3
Streptomyces aureoverticillatus NAT1
Streptomyces sp. RI18 NAT/bezG
Streptomyces autolyticus NAT1
Streptomyces malaysiensis NAT2
Gordonia malaquae NAT1
Nocardia speluncae NAT1
Nocardia cyriacigeorgica NAT1
Nocardia sienata NAT1
Nocardia testacea NAT1
Streptomyces sp. NRRL F-4489 NAT1
Streptomyces noursei NAT1
Streptomyces yunnanensis NAT1
Streptomyces albofaciens NAT1
Streptomyces peucetius NAT1
Streptomyces capuensis NAT1
Streptomyces lavendulae subsp. lavendulae NAT1
Streptomyces rimosus NAT1
Streptomyces sp. WAC 06783 NAT1
Bradyrhizobium oligotrophicum NAT1
Bradyrhizobium oligotrophicum NAT2
Pseudonocardia hydrocarbonoxydans NAT1
Nannocystis exedens NAT1
Sphingosinicella sp. NAT1
Massilia namucuonensis NAT1
Bosea vaviloviae NAT1
Bosea lathyri NAT1
Bosea sp. NAT1
Mycolicibacterium gadium NAT1
Mycobacterium bohemicum NAT1
Mycobacterium lentiflavum NAT1
Williamsia limnetica NAT1
Williamsia marianensis NAT1
Williamsia muralis NAT1
Pseudonocardia endophytica NAT1
Pseudonocardia sediminis NAT1
Pseudonocardia alni NAT1
Pseudonocardia sp. AL041005-10 NAT1
Pseudonocardia ammonioxydans NAT1
Pseudonocardia autotrophica NAT1
Pseudonocardia saturnea NAT1
Halostella salina NAT1
Pontibacillus halophilus NAT1
Staphylococcus felis NAT1
Paenibacillaceae bacterium NAT2
Flagellimonas pacifica NAT1
Geitlerinema sp. NAT1
Staphylospora marina NAT1
Thermoactinomyces daqus NAT1
Vibrio nigripulchritudo NAT1
Angiococcus disciformis NAT/nubG
Melghirimyces algeriensis NAT1
Quasibacillus thermotolerans NAT1
Bacillus anthracis NAT3
Bacillus thuringiensis NAT3
Bacillus cereus NAT2
Bacillus bombysepticus NAT1
Bacillus mycoides NAT1
Microlunatus phosphovorus NAT1
Allokutzneria albata NAT1
Longispora albida NAT2
Amycolatopsis cihanbeyliensis NAT2
Streptomyces collinus NAT1
Streptomyces kanamyceticus NAT2
Streptomyces platensis NAT/pmC
Streptomyces griseoruber NAT/orf2
Micromonospora sp. B006 NAT1
Micromonospora sp. B006 NAT2
Streptomyces albus subsp. chlorinus strain LW030448 NAT/nybK
Streptomyces sp. F001 NAT2
Streptomyces sp. F001 NAT3
Streptomyces aureus NAT/colC2
Saccharothrix espanaensis NAT2
Streptomyces nodosus NAT/asuC2
Streptomyces hygroscopicus NAT/gdmF
Actinosynnema mirum NAT3
Actinosynnema pretiosum NAT2
Frankia sp. NAT1
Pseudonocardia oroxyli NAT1
Saccharopolyspora erythraea NAT2
Micromonospora sp. HK160111 NAT/mas10
Amycolatopsis orientalis NAT3
Amycolatopsis alba NAT1
Actinosynnema mirum NAT2
Actinosynnema pretiosum NAT3
Actinosynnema pretiosum NAT1
Actinosynnema pretiosum subsp. auranticum NAT/asm9
Streptomyces filamentosus NAT2
Streptomyces malaysiensis NAT4
Streptomyces sp. HKI0576 NAT/divN
Streptomyces rapamycinicus NAT2
Streptomyces violaceusniger NAT3
Streptomyces malaysiensis NAT3
Streptomyces sp. LZ35 NAT/hgcF
Streptomyces achromogenes NAT/rubF
Streptomyces sp. CS NAT/natF
Streptomyces spectabilis NAT4
Streptomyces spectabilis strain CCTCC M2017417 NAT/stvF
Salinispora arenicola CNS-205 NAT/Sare 1251
Streptomyces leeuwenhoekii NAT/cxmF
Amycolatopsis vancoresmycina NAT5
Amycolatopsis mediterranei NAT3
Amycolatopsis sp. NAT/rmpF
Cluster types
PKS
PKS-NRPS hybrids
Other
Terpene
NRPS
Thiopeptide
Other NRPS-hybrids
